# Predictive biases in emotional perception: differential influence of mood and affective primes in individuals with and without mood disorders

**DOI:** 10.1093/nc/niag072

**Published:** 2026-10-31

**Authors:** Alexane Leclerc, Ladislas Nalborczyk, Louise Kauffmann, Yann Leprince, Josselin Houenou, Lucie Berkovitch, Pauline Favre

**Affiliations:** INSERM U955-IMRB, Translational NeuroPsychiatry Team, University Paris-Est Créteil, 8 rue du Général Sarrail, 94010 Créteil, France; UNIACT, INPULSE Team, NeuroSpin, CEA Paris-Saclay, Building 145, 91191 Gif-sur-Yvette, France; Fondation FondaMental, Albert Chenevier, Hospital Psychiatry department, 40 rue de Mesly, 94000 Créteil, France; LPC, CNRS, Aix-Marseille University, 3 place Victor Hugo, 13331 Marseille, France; LPNC, CNRS, University Grenoble Alpes, University Savoie Mont Blanc, Michel Dubois Building, 1251 rue des Université, 38058 Grenoble, France; UNIACT, NeuroSpin, CEA, Université Paris-Saclay, Building 145, 91191 Gif-sur-Yvette, France; INSERM U955-IMRB, Translational NeuroPsychiatry Team, University Paris-Est Créteil, 8 rue du Général Sarrail, 94010 Créteil, France; UNIACT, INPULSE Team, NeuroSpin, CEA Paris-Saclay, Building 145, 91191 Gif-sur-Yvette, France; Fondation FondaMental, Albert Chenevier, Hospital Psychiatry department, 40 rue de Mesly, 94000 Créteil, France; Department of Psychiatry, University of Melbourne, Parkville, 3010 Victoria, Australia; Neuromodulation Institute, GHU Paris Psychiatry and Neurosciences, Sainte-Anne Hospital, 12 rue Cabanis, 75014 Paris, France; Cognitive Neuroimaging Unit, NeuroSpin, INSERM-CEA, University Paris-Saclay, Building 145, 91191 Gif-sur-Yvette, France; INSERM U955-IMRB, Translational NeuroPsychiatry Team, University Paris-Est Créteil, 8 rue du Général Sarrail, 94010 Créteil, France; UNIACT, INPULSE Team, NeuroSpin, CEA Paris-Saclay, Building 145, 91191 Gif-sur-Yvette, France; Fondation FondaMental, Albert Chenevier, Hospital Psychiatry department, 40 rue de Mesly, 94000 Créteil, France

**Keywords:** affective priming, predictive processing, emotional perception, bipolar disorder, depression

## Abstract

Predictive processing models perception as an interplay between top-down predictions and bottom-up sensory inputs. When signals are ambiguous, perception increasingly relies on prior expectations. In emotional contexts, mood and affective primes may act as endogenous and exogenous predictions, biasing emotional perception, particularly in mood disorders, where predictions may become overly strong or unstable. Eighty-four participants (53 healthy controls, HC; 31 with self-reported mood disorders) completed an online affective priming task on unambiguous and ambiguous (morphs) facial expressions, preceded by masked or unmasked faces. Participants rated target emotions and completed mood questionnaires. Principal Component Analysis extracted latent mood dimensions. Drift Diffusion Modeling (DDM) estimated cognitive processes underlying ambiguous face perception. In HC and bipolar disorder (BD), happy primes increased positive morph categorization, while in depression (DEP), masked sad primes increased negative categorizations more than in HC and BD. Masked primes accelerated responses to morphs in HC, while BD showed faster responses regardless of priming type, with stronger facilitation than HC and DEP. No reaction time priming effects were observed in DEP. DDM revealed pre-decisional biases induced by primes, while mood only subtly influenced evidence accumulation. Findings suggest group-specific predictive biases: HC used masked primes to resolve emotional ambiguity, BD showed stronger reliance on external cues regardless of priming type, and DEP showed reduced sensitivity to primes alongside a selective negativity categorization bias. These biases may underlie core clinical features of mood disorders and may point to new targets for interventions aiming to restore adaptive emotional inference.

## Introduction

Biases in emotional perception are a core feature of mood disorders and may contribute to their onset, maintenance, and relapse (Penton-Voak et al. 2017). Understanding the cognitive mechanisms underlying these biases is crucial to improve diagnostic, and prognostic outcomes, as well as to develop targeted treatment strategies. A promising framework for understanding how individuals process (ambiguous) emotional information is predictive processing (PP), which models perception as a combination of top-down predictions from prior experience and bottom-up sensory inputs (Rao and Ballard 1999). When sensory information is ambiguous, prior predictions will further be used to infer the content of perception (Scocchia et al. 2014).

This framework extends to emotional face perception. Indeed, contextual cues, such as emotional background scenes, prosody, or sentences, and emotional prime faces, whether briefly presented (masked) or consciously perceived (unmasked), can facilitate recognition of congruent expressions (Neumann and Lozo 2012, Rohr et al. 2012, Pell et al. 2022, Bryce et al. 2024, Pullinen et al. 2026) and bias perception of ambiguous targets toward congruent interpretations (Haneda et al. 2003, Lu et al. 2011). These effects may reflect a pre-activation of emotion categories, shaping expectations about which emotions are more likely to appear.

On top of affective cues, mood exerts a top-down influence on emotional perception (Forgas 2017), possibly shaping prior predictions that facilitate processing of mood-congruent information and bias interpretation of emotional ambiguity. Transient, experimentally induced mood states (*e.g. via* emotional images or autobiographical recall) influence subtle or ambiguous facial expression toward congruent interpretations (Schmid and Schmid Mast 2010, Qiao-Tasserit et al. 2017, Trilla et al. 2021).

Endogenous and long-lasting negative mood states are similarly associated with negative biases in interpreting morphed expressions in healthy individuals (Qiao-Tasserit et al. 2017). Such biases may be amplified in mood disorders like major depressive disorder (MDD) and bipolar disorder (BD), which are marked by mood fluctuations of high amplitude and variable duration. According to PP, depression involves overly-rigid negative predictions which are insufficiently updated by disconfirming evidence, producing a range of negative perception and beliefs, and perpetuating negative biases (Kube et al. 2020, Bottemanne et al. 2022, 2023, Ramos-Grille et al. 2022).

Previous affective priming studies are limited by heterogeneous samples, few repetitions per conditions, and by the use of identical stimuli for primes and targets, potentially yielding automatic stimulus–response mapping (Abrams and Greenwald 2000, Damian 2001). Replication and extension are needed, particularly using ambiguous faces morphed between two distinct expressions (i.e. happiness and sadness), which may carry higher perceptual uncertainty than morphs between a neutral and a single emotional expression, and thus enhance sensitivity to affective PP. Moreover, comparing masked and unmasked priming may also help to dissociate automatic from more deliberate processing respectively, with unmasked primes—being fully visible—expected to produce stronger predictive effects on emotional perception (Francken et al. 2011).

This study investigates how mood and affective cues influence emotional perception in individuals with and without mood disorders. Healthy controls (HC) and individuals with self-reported BD and depression (DEP) completed an affective priming task on ambiguous and unambiguous faces. Happy, sad, or morphed (i.e. both happy and sad) target faces were preceded by masked or unmasked prime faces (happy, sad, morphed, or none), and participants rated the target’s emotion and completed mood questionnaires. Moreover, to better understand the latent cognitive processes underlying decision-making under emotional ambiguity, we complemented behavioral analyses with Drift Diffusion Modeling (DDM). DDM allowed us to model how participants decide between positive and negative interpretations of ambiguous emotional stimuli by jointly accounting for response choices and RTs on a trial-by-trial basis (see Supplementary Method S7). In particular, DDM enables the estimation of drift rate, reflecting the speed and direction of evidence accumulation toward a positive or negative interpretation of ambiguous morphs, and the starting point, reflecting pre-decisional biases favoring one response over the other. From a PP perspective, mood and emotional primes may bias emotional perception either by influencing the evaluation of incoming sensory evidence (reflected in changes in drift rate) or by shaping prior expectations before evidence accumulation begins (reflected in shifts in starting point). Consistent with this framework, previous work has shown that mood manipulations can induce latent prediction biases detectable with DDM even in the absence of overt response differences (White et al. 2018).

Based on the PP framework, we hypothesized that mood and affective primes would influence emotional perception.

*(1) Priming effects on reaction times (RTs) to unambiguous emotional targets (happy/sad). (1a)* We expected faster RTs when targets were preceded by emotionally congruent primes (e.g. faster RTs for happy targets following happy primes), in both masked and unmasked trials. *(1b)* These effects should be stronger in BD than in HC and DEP. *(1c)* Based on prior findings on syntactic priming (e.g. Berkovitch and Dehaene 2019), we further predicted stronger priming in unmasked than masked trials.

*(2) Effects of mood and primes on the categorization of ambiguous targets. (2a)* We hypothesized that participants’ mood, as measured by a principal component analysis, should bias morph categorization in a mood-congruent manner. *(2b)* Emotional primes (happy or sad versus control) would influence morph perception toward more positive or negative interpretations in both masked and unmasked trials. *(2c)* These prime-induced categorization biases should be stronger in BD than in HC*. (2d)* In DEP, we expected a negativity bias: an enhanced influence of sad primes versus HC, and a reduced influence of happy primes relative to HC and BD. *(2e)* Finally, we further predicted stronger priming effects unmasked than masked trials across groups.

*(3) Priming effects on RTs to ambiguous targets. (3a)* We expected faster RTs for morphed targets when the prime was congruent with the participant’s categorical response (*e.g.* faster RTs for morphs categorized as positive following happy primes). *(3b)* These effects should be larger in BD than in HC and DEP. *(3c)* We further expected larger RT priming effects in unmasked compared to masked conditions.

*(4) Effects of mood and primes on perceptual decision making for ambiguous targets (DDM)*. *(4a)* We predicted that a more positive mood would be associated with both higher drift rates and a starting point biased toward positive responses when evaluating morphed faces. *(4b)* Happy primes (versus control) should increase drift rate toward positive interpretations and shift the starting point toward the positive response boundary, with sad primes inducing the opposite pattern. *(4c)* These prime-induced shifts should be stronger in BD than in HC. *(4d)* In DEP, we expected the same negativity bias: a selective influence of sad primes on drift rate and starting point relative to HC, and a reduced sensitivity to happy primes compared to the other groups. No specific effects were hypothesized for decision threshold or non-decision time.

## Methods

### Participants

A total of 107 French-speaking participants took part in the experiment, including 67 HC (29 women, mean age = 32, standard deviation (SD) = 9.80), 20 with a self-reported BD diagnosis (8 women, mean age = 31, SD = 9.33), and 20 with self-reported DEP (9 women, mean age = 31, SD = 8.09). Patients reported having received a formal diagnosis of BD or DEP, and indicated that their condition was ongoing at the time of the study. Recruitment was conducted *via* Prolific (www.prolific.com), and we included all French-speaking BD participants who met our pre-screening criteria and were available on the platform at the time of data collection. See Supplementary Method S1 for sample size calculation, and Supplementary Methods S2 and S3 for recruitment and patients’ pre-screening. Participants were excluded if more than 5% of their trials showed invalid prime durations (< 20 ms or > 45 ms), or if performance on unambiguous emotional targets was below 2 SD from the mean. Given the online nature of the experiment, conservative RT criteria were applied: participants with mean RTs outside ±2 SD were excluded, and trials with RTs > 3000 ms or outside ±3 SD (computed by target type and group) were removed. See Supplementary Method S3 for more information on exclusion criteria, and Table S1 for demographics. In total, 53 HC and 31 patients—including 14 BD and 17 DEP—were included in the final analysis. The study was approved by the Research Ethics Committee of Paris-Saclay University (reference: CER-Paris-Saclay-2023-029A1).

### Stimuli and experimental design

Stimuli were created using happy and sad facial expressions of 24 individuals (12 females) from the FACES database (Ebner et al. 2010), resulting in a total of 72 stimuli (24 happy, 24 sad, 24 morphs). Participants were presented with emotional target faces (happy, sad, or morphed) while being primed with a masked or unmasked happy, sad, morph prime face, or a control condition (blank oval). Their task was to rate as quickly as possible the valence of the emotion perceived on the target by pressing with their index and middle finger on the keys [v] and [b] representing ‘Negative’ or ‘Positive’ (counterbalanced across participants). The next trial started once participants responded. Prime-target pairings differed in identity, matched gender, and avoided consecutive repetition. The content of a trial is depicted in Fig. 1. In total, there were 864 randomized trials, evenly split across conditions. For details, refer to Supplementary Method S3.

**Figure 1:**
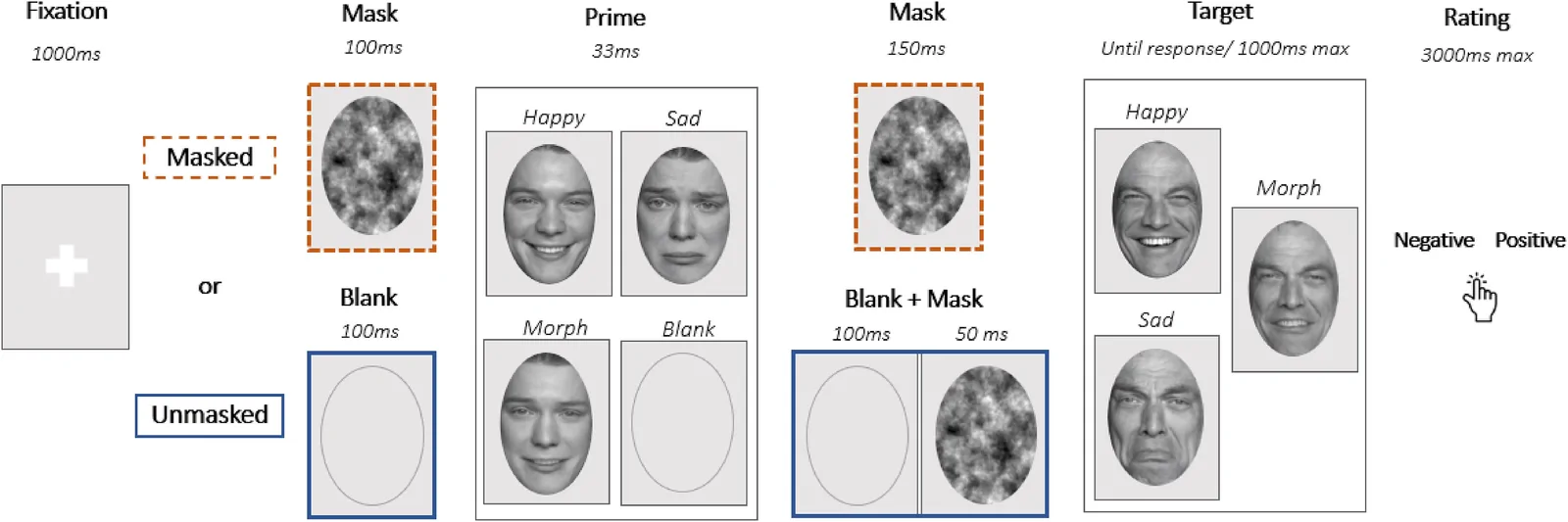
Experimental design. Participants were presented with a target face (happy, sad or morph) for 1000 ms, preceded by a masked or unmasked 33 ms prime (happy, sad, morph face or a blank oval). Their task was to rate as fast as possible the perceived valence of the targets by pressing with their index and middle fingers on the keys [v] and [b] representing ‘negative’ or ‘positive’ (counterbalanced across participants). The next trial started once participants responded, or after a 3000 ms timeout. In masked trials, the prime was preceded by a 100 ms mask and directly followed by another 150 ms mask to avoid retinal persistence. In unmasked trials, the prime was preceded and followed by a 100 ms blank, and a shorter mask of 50 ms. Due to copyright restrictions, the depicted face identities do not correspond to the ones used in the study.

### Procedure

Participants accessed the study link *via* Prolific, provided informed consent, and underwent a screen refresh-rate check (57–63 Hz required). Then, they completed two mood questionnaires: the Positive and Negative Affect Scale (PANAS; Watson et al. 1988) and Multidimensional Assessment of Thymic States scales (MAThyS; Henry et al. 2008a). After instructions and 12 practice trials, participants completed the main task, divided into four blocks of 216 trials with one minute breaks in between. At the end, subjects completed a visibility check assessing prime perception with 72 additional masked trials, equally distributed among the four primes. Participants indicated prime visibility (‘Seen’/‘Not Seen’) and categorized prime emotion (‘Positive’/‘Negative’).

### Data analysis

Data were analyzed using R statistical software (version 4.3.2; R Core Team 2023).

To obtain a single mood score per participant, we performed a Principal Component Analysis (PCA) on the 47 items from the two mood questionnaires and retained the principal component (PC) which explained most variance (PC1) as a proxy of mood (Ji et al. 2021, Lee et al. 2024, Moujaes et al. 2024). PC1 reflects a latent dimension of the mood questionnaires (*e.g.* positive affect, or reactivity), with some items contributing more strongly than others. If items related to positive emotions show high positive loadings and items related to negative emotions show negative loadings, PC1 could be interpreted as a latent positive affect dimension.

RTs were analyzed using Bayesian generalized linear mixed-effects models with a log-normal likelihood, implemented in the *brms* package (Bürkner 2017). Separate models were estimated for unambiguous and ambiguous emotional targets. For both models, Congruence (Congruent, Incongruent), Priming Type (Masked, Unmasked), Group (HC, BD, DEP), and their interactions were included as fixed effects. In the unambiguous model, Target Valence (Happy, Sad) was included as an additional covariate. In the ambiguous model, Response Valence (Positive, Negative) was included instead. Random effects included by-participant intercepts and slopes for Congruence × Priming Type, as well as by-target intercepts. For analyses of unambiguous targets, only correct trials were included.

Categorization of ambiguous morphs as Positive or Negative was analysed using a Bayesian generalized linear mixed-effects model with a Bernoulli likelihood and logit link using the *brms* package (Bürkner 2017). The emotional expression of the Prime (Happy, Sad, Control), Priming Type (Masked, Unmasked), Group (HC, BD, DEP), Mood (PC1), and their interactions were included as fixed effects. Random effects included by-participant intercepts and slopes for Prime × Priming Type, as well as by-target intercepts.

To further investigate the effect of mood and primes on emotional ambiguity resolution, we fitted a multilevel Bayesian drift diffusion model (Fig. 2) *via brms* (Bürkner 2017). Predictors included Prime (Happy/Sad/Control), Priming type (Masked/Unmasked), Mood (PC1), and their interactions, with random intercept and varying slope for the effect of Prime and Priming type per participant, and by-target intercepts.

**Figure 2:**
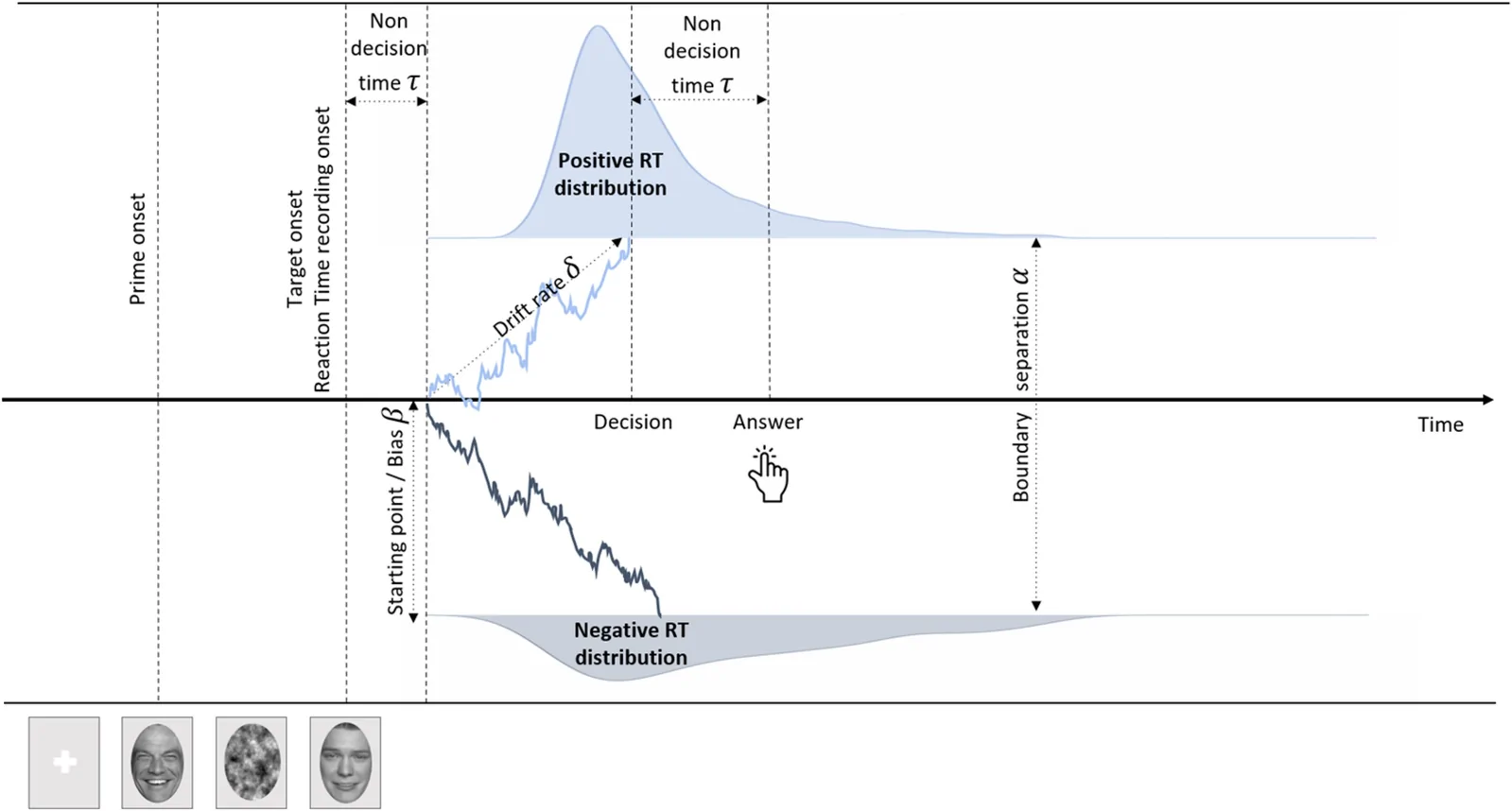
Parameters and assumptions of the DDM. The trial begins with a fixation cross, followed by the prime, a mask, and the target stimulus (after which reaction times are recorded). In its simplest (vanilla) version, the model estimates four parameters: non-decision time (sensory and motor processes), starting point (initial bias toward positive or negative answer, hypothesized to be influenced by mood and the primes), drift rate (speed of evidence accumulation toward a decision), and the decision threshold (evidence required to decide). Figure adapted with permission from Lacroix et al. (2021). Due to copyright restrictions, the depicted face identities do not correspond to the ones used in the study.

Across models, for the effects of interest, we report posterior means with 95% credible intervals (CrIs) and Bayes Factors (BF_10_), interpreted continuously but also provide thresholds for easier interpretation following Wagenmakers et al. (2018): anecdotal (1–3), moderate (3–10), strong (10–30), very strong (30–100). We also report the proportion of the posterior distribution falling within the region of practical equivalence (ROPE) when evidence is ambiguous (1 < BF_10_ < 3). See Supplementary Method S6 for more details.

To assess prime visibility and discrimination between happy and sad primes, we computed signal detection theory (SDT) *d’* scores (Green & Swets, 1966). Evidence for above-chance discrimination within each group, and for differences in discriminability between groups, was quantified with 95% CrIs and BF_10_. We further tested whether the magnitude of RT priming effects scaled with individual differences in *d′*.

Full analysis pipelines—including PCA methods, RT and categorization models, DDM specifications, and visibility task methods—are provided in Supplementary Methods S5–S8.

## Results

To facilitate navigation through the Results section, Supplementary Table S2 summarizes each hypothesis and its main findings, indicating whether each prediction was supported, partially supported, or not supported by the data.

### Principal component analysis

PCA on mood questionnaires revealed four significant PCs (*P* < .05 across all permutations) explaining 22%, 16%, 7%, and 5% of variance respectively. PC1 was retained as a proxy of mood (Fig. 3). It describes a single affective dimension running from negative to positive affect: positive-affect items (*e.g.* enthusiastic, energetic, active) loaded positively and negative-affect items (*e.g.* nervous, sadness, anxiety, irritability) negatively, with loadings of comparable magnitude at both poles. PC2 captured high emotional intensity and mixed-valence states. PC3 reflected reactivity, distractibility, and hypersensitivity, while PC4 related to internal tension and sensory-affective excitability. Mood questionnaires scores by group can be found in Supplementary Tables S3. Detailed feature contributions and group comparisons for PC1–PC4 are provided in Supplementary Tables S4–S7, and Figs S1–S3. Given its interpretability as a reactivity index, we tested whether PC3 was associated with RT priming scores; however, no significant correlations emerged (all *P* > .05; see Supplementary Method S8, Supplementary Result S1, Figs S4–S7).

**Figure 3:**
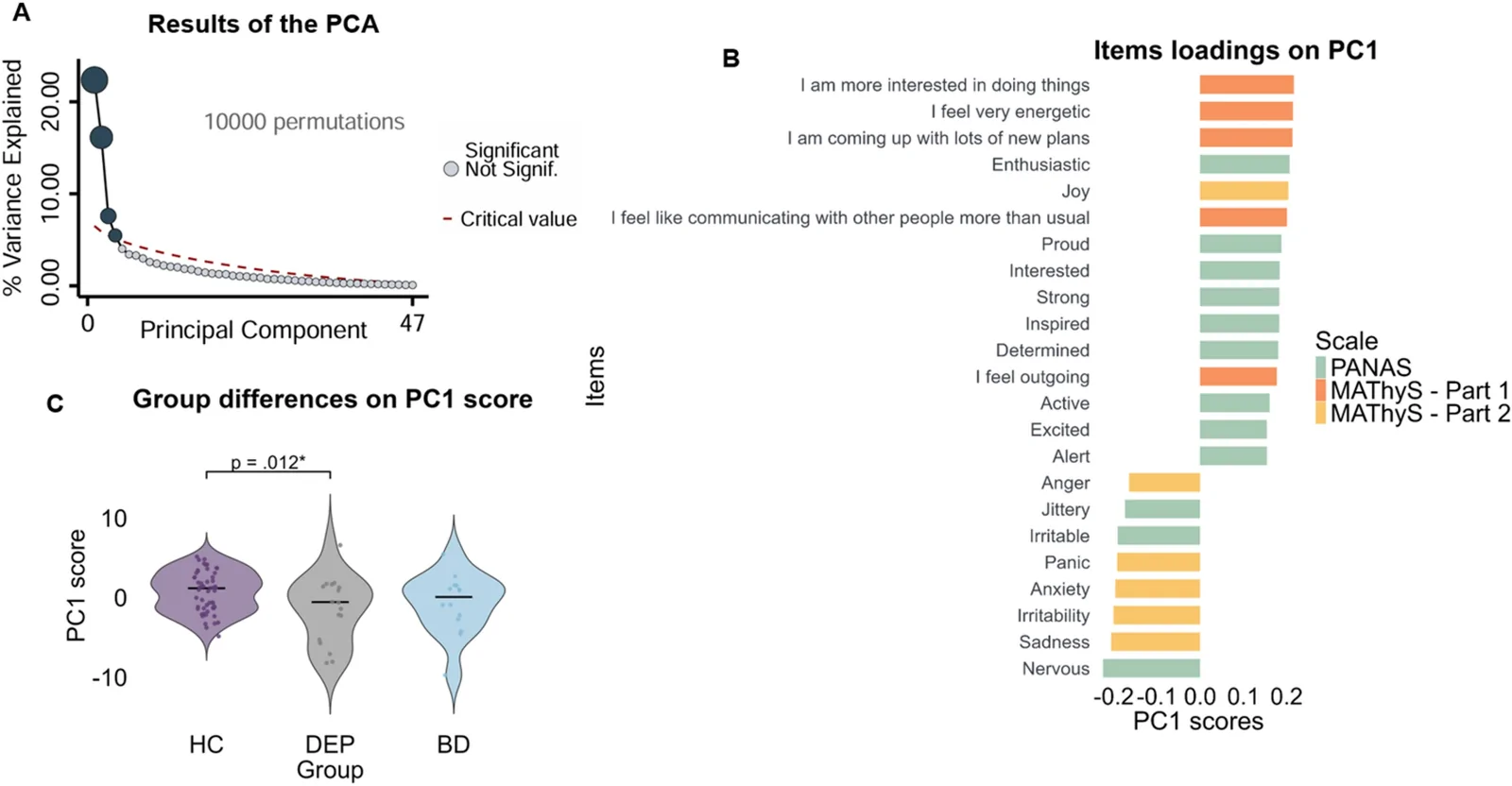
Principal component analysis of mood questionnaire items. **A.** Results of the PCA. Percentage of variance explained by each PC, based on 10 000 permutations. Dots represent observed eigenvalues; the dashed line indicates the critical value for statistical significance. The four components above this threshold were retained. **B.** Items loadings on PC1, limited to those with absolute values > 0.15 for clarity. Loadings represent the contribution of each item to the principal component. Items are color-coded by questionnaires (PANAS, MAThyS—Part 1, Part 2). PC1 describes a single affective dimension running from negative to positive affect. **C.** Group differences in PC1 scores across groups (BD, DEP, HC). Each dot represents PC1 score for one participant, and the vertical bar indicates the group mean. Violin plots display the distribution and density of scores across participants. Statistically significant effects are denoted by *P* < .05 (*P* < .05 ^*^, *P* < .01 ^**^, *P* < .001 ^***^).

*(1) Priming effects on RTs to unambiguous emotional targets (happy/sad)*

*(1a)* In masked trials, we found extremely strong evidence for faster RTs following congruent versus incongruent primes in HC (β = −0.049, 95% CrI [−0.06, −0.04], BF_10_ = 9.37 × 10^6^), BD (β = −0.049, 95% CrI [−0.07, −0.03], BF_10_ = 182.48), and in DEP (β = −0.041, 95% CrI [−0.06, −0.02], BF_10_ = 87).

In unmasked trials, we found extremely strong evidence for faster RTs following congruent versus incongruent primes HC (β = −0.031, 95% CrI [−0.04, −0.02], BF_10_ = 2.59 × 10^3^) and BD (β = −0.045, 95% CrI [−0.07, −0.02], BF_10_ = 97.64). In contrast, the model showed moderate evidence in favor of the null hypothesis in DEP (β = −0.019, 95% CrI [−0.04, 0], BF_10_ = 0.33).

*(1b)* Regarding between-group differences, there was moderate evidence for no differences between groups in masked (all 0.11 < BF_10_ < 0.13) and unmasked trials (all 0.19 < BF_10_ < 0.4).

*(1c)* Comparing masked and unmasked trials, the model indicated that H_1_ (congruency effect) was 2.5 times more likely than the null in HC (β = −0.018, 95% CrI [0, 0.03], BF_10_ = 2.49, with 40.57% of the posterior within the ROPE). Conversely, there is very strong evidence for no difference in priming effects between masked and unmasked trials in BD (β = 0.004, 95% CrI [−0.020, 0.03], BF_10_ = 0.09), but no conclusive evidence for a difference in DEP (β = 0.023, 95% CrI [0, 0.05], BF_10_ = 0.58).

Finally, results provided moderate evidence for the absence of a differential congruence effect between happy and sad targets across masked and unmasked trials and groups (all 0.1 < BF_10_ < 0.39). The proportion of correct responses for the same conditions is provided in Supplementary Table S8. See Supplementary Tables S9–S13, and Fig. 4 for results across groups, and Supplementary Fig. S8 for model diagnostics. Mean RTs and standard deviations for happy and sad targets by group, prime congruence, and priming type are reported in Table 1.

**Figure 4:**
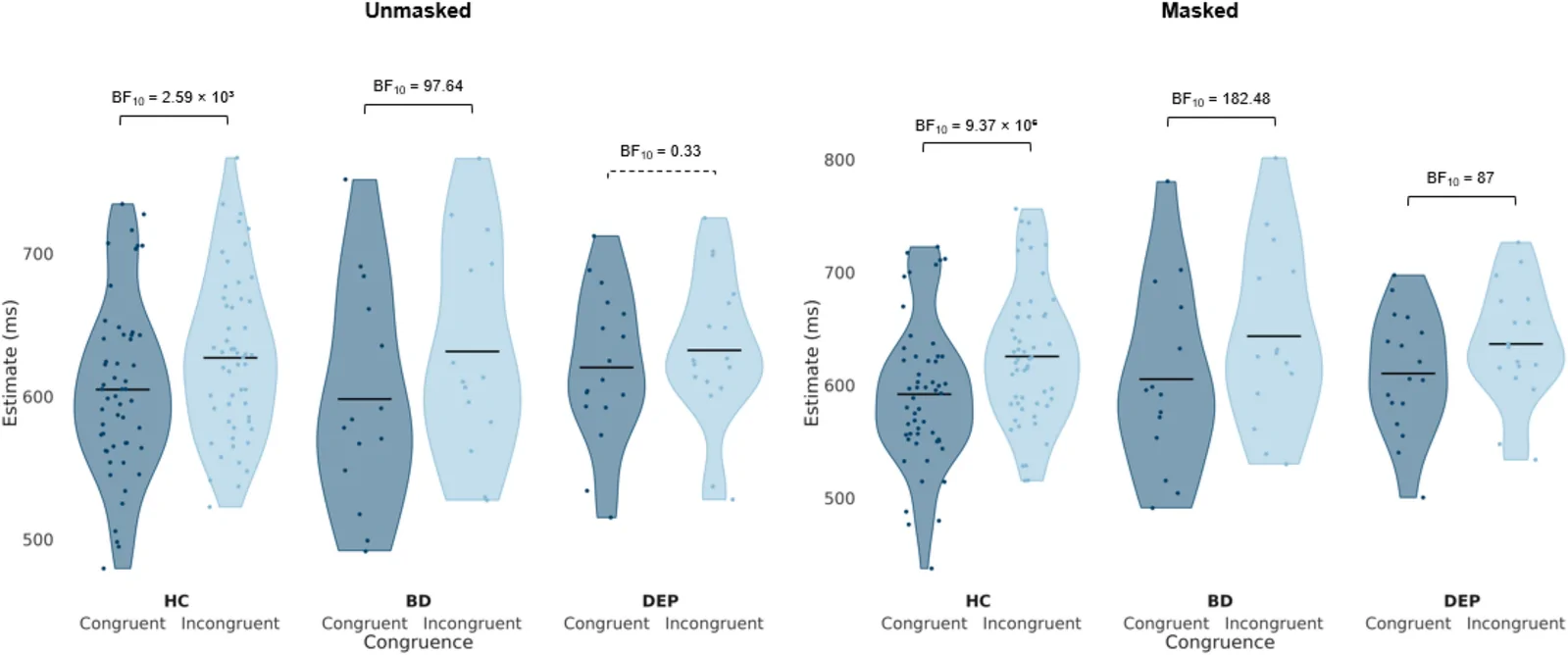
Posterior mean estimates of RTs to unambiguous emotional targets (happy and sad) as a function of prime congruence (congruent, incongruent) and priming type (masked, unmasked) in HC, BD patients, and DEP patients, derived from Bayesian generalized linear mixed-effects models with log-normal likelihood. Each dot represents the estimated posterior mean RT for one participant, and the horizontal bar indicates the group mean. Violin plots display the distribution and density of estimated RTs across participants. BF_10_ values reflect the strength of evidence for the congruence effect; brackets indicate evidence in favor of the alternative hypothesis, while dashed brackets indicate evidence in favor of the null hypothesis.

**Table 1:** Mean RTs (M, in milliseconds) and standard deviations (SD) for unambiguous (happy and sad) and ambiguous (morph) targets, as a function of prime congruence (congruent/incongruent) and priming type (masked, unmasked) in HC, BD patients, and DEP patients

**Group**	**Target**	**Prime Congruence**	**Priming Type**	**M (ms)**	**SD**
**HC (*n* = 53)**	**Happy and Sad**	**Congruent**	**Masked**	596.27	2.24
		**Incongruent**		624.69	2.09
		**Congruent**	**Unmasked**	610.36	2.24
		**Incongruent**		626.83	2.10
	**Morphs**	**Congruent**	**Masked**	702.79	10.90
		**Incongruent**		730.42	9.75
		**Congruent**	**Unmasked**	716.70	10.65
		**Incongruent**		722.53	9.62
**BD (*n* = 14)**	**Happy and Sad**	**Congruent**	**Masked**	610.14	5.41
		**Incongruent**		640.43	5.02
		**Congruent**	**Unmasked**	614.15	5.29
		**Incongruent**		635.96	4.90
	**Morphs**	**Congruent**	**Masked**	684.58	21.05
		**Incongruent**		781.77	23.76
		**Congruent**	**Unmasked**	717.77	23.73
		**Incongruent**		773.33	32.41
**DEP (*n* = 17)**	**Happy and Sad**	**Congruent**	**Masked**	611.15	4.33
		**Incongruent**		633.77	3.89
		**Congruent**	**Unmasked**	626.54	4.59
		**Incongruent**		635.10	4.08
	**Morphs**	**Congruent**	**Masked**	741.29	19.31
		**Incongruent**		713.12	15.27
		**Congruent**	**Unmasked**	740.60	22.33
		**Incongruent**		744.07	18.22

*(2) Effects of mood and primes on the categorization of ambiguous targets.*

*(2a)* We found no conclusive evidence for an effect of mood (as indexed by PC1) on morph categorization across groups (β = 0.139, 95% CrI [0.00, 0.28], BF_10_ = 0.9). Exploratory analyses examined this effect within each group. In HC and DEP, we found no conclusive evidence for an effect of mood on morph categorization (HC, β = 0.197, 95% CrI [0.00, 0.40], BF_10_ = 1.32, ROPE = 42.88%; DEP, β = 0.211, 95% CrI [−0.02, 0.43], BF_10_ = 0.93). In BD, we found moderate evidence for an absence of effect (β = 0.009, 95% CrI [−0.27, 0.28], BF_10_ = 0.2). See Supplementary Tables S14–S16, and Fig. 5 for results across groups.

**Figure 5:**
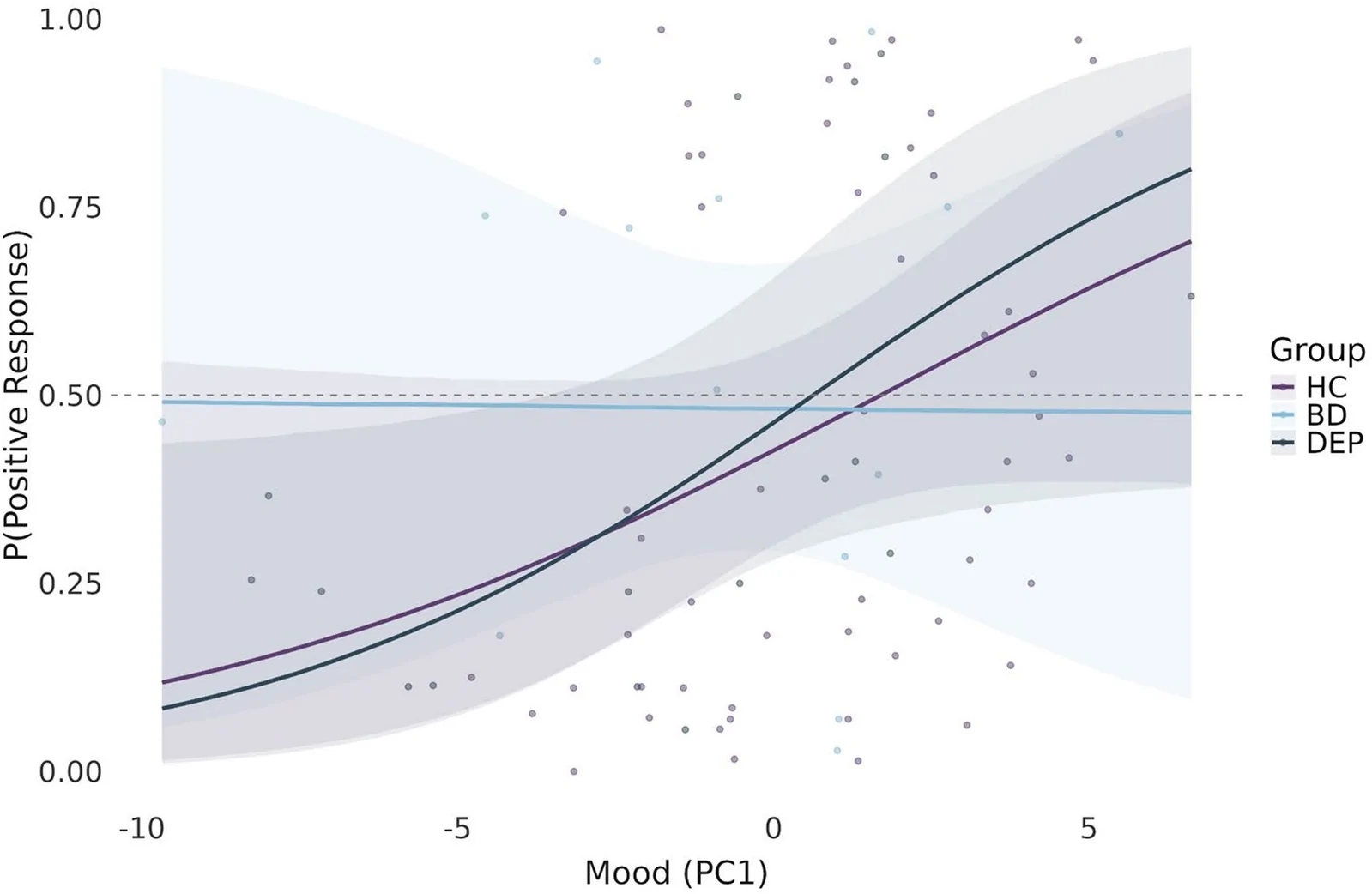
Predicted probability of a positive response to ambiguous facial expressions as a function of mood (PC1) for HC, BD, and DEP, derived from a Bayesian generalized linear mixed-effects model (Bernoulli likelihood). Each dot represents a participant’s average proportion of positive responses plotted against their mood score (PC1). Lines show the predicted probability of a positive response and shaded areas indicate the 95% credible intervals. The dashed horizontal line indicates chance level (*P* = 0.50).

*(2b)* In masked trials, we found anecdotal evidence for more positive categorization of morphs following positive versus no primes in HC, with the data being 2.22 times more likely under the alternative than the null (β = −0.228, 95% CrI [−0.42,-0.03], BF_10_ = 2.22, with 32.02% of the posterior within the ROPE). In BD, we found strong evidence for an effect of happy primes versus no prime on morph categorization (β = −0.528, 95% CrI [−0.90,-0.15], BF_10_ = 11.24). However, in DEP we found moderate evidence for the null hypothesis (β = −0.085, 95% CrI [−0.26,-0.43], BF_10_ = 0.25). Regarding sad primes, we found moderate evidence for the absence of an effect on sad interpretations of morphs in HC and BD (HC: β = −0.008, 95% CrI [−0.20, 0.18], BF_10_ = 0.18; BD: β = −0.148, 95% CrI [−0.51, 0.19], BF_10_ = 0.32). In contrast, results provided moderate evidence for more negative categorization of morphs following sad compared to no primes in DEP (β = 0.412, 95% CrI [0.09, 0.74], BF_10_ = 4.5).

In unmasked trials, we found extremely strong evidence for more positive categorization of morphs following positive versus no primes in HC (β = −0.481, 95% CrI [−0.68,-0.28], BF_10_ = 2.33 × 10^3^) and BD (β = −0.779, 95% CrI [−1.15,-0.40], BF_10_ = 325.23). However, in DEP we found no conclusive evidence in favor of H_1_ (β = −0.354, 95% CrI [−0.69,-0.02], BF_10_ = 1.82, ROPE = 15.32%). Regarding sad primes, we found no conclusive evidence for more negative categorization of morphs following sad versus no primes in HC (β = −0.207, 95% CrI [−0.40,-0.02], BF_10_ = 1.63, ROPE = 39.07%), and moderate evidence for an absence of effect in BD (β = −0.131, 95% CrI [−0.48,0.22], BF_10_ = 0.3) and DEP (β = −0.158, 95% CrI [−0.49,0.17], BF_10_ = 0.33).

*(2c–d)* Regarding between-group differences, in masked trials, we found moderate evidence for a stronger effect of happy primes (versus control) on positive morph categorization in BD compared to DEP (β = −0.613, 95% CrI [−1.11, −0.11], BF_10_ = 5.94). For sad primes, we found moderate evidence for a stronger effect of sad primes (versus control) on negative morph categorization in DEP compared to HC (β = −0.42, 95% CrI [−0.78, −0.06], BF_10_ = 4.11) and BD (β = −0.56, 95% CrI [−1.02, −0.07], BF_10_ = 4.49).

In unmasked trials, we found no conclusive evidence in favor of H_1_ for group differences in the effect of happy primes (all 0.43 < BF_10_ < 1.3). However, results provided moderate evidence for the absence of group differences in the effect of sad primes (versus control) on negative morph categorization (all 0.3 < BF_10_ < 0.37).

*(2e)* Comparing masked and unmasked trials, the model revealed no conclusive evidence in favor of H_1_ for a differential effect of positive primes on morph categorization in HC (β = −0.253, 95% CrI [0,0.50], BF_10_ = 1.9, with 27.96% of the posterior within the ROPE) and BD (β = −0.252, 95% CrI [−0.21,0.70], BF_10_ = 0.59). In DEP, results provided anecdotal evidence for a stronger effect of happy primes on positive morph categorization in unmasked compared to masked trials, with the data being 2.56 times more likely under the alternative than the null (β = 0.439, 95% CrI [0.02,0.85], BF_10_ = 2.56, with 10.87% of the posterior within the ROPE). Regarding sad primes, we found no conclusive evidence in favor of H_1_ for a difference between masked and unmasked trials in HC (β = 0.199, 95% CrI [−0.04,0.45], BF_10_ = 0.9), and moderate evidence for an absence of effect in BD β = −0.017, 95% CrI [−0.45,0.42], BF_10_ = 0.31. In contrast, we found strong evidence for a stronger effect of masked compared to unmasked sad prime (as compared to control) on negative morph categorization in DEP (β = 0.57, 95% CrI [0.16,0.99], BF_10_ = 11.53). See Supplementary Fig. S9 for model diagnostics, and Table 2, Supplementary Tables S17–S19, and Fig. 6 for results across groups.

**Table 2:** Probability of a positive response to ambiguous morph targets, as a function of priming type (masked, unmasked), in HC, patients with BD, and patients with DEP

**Group**	**Priming Type**	**Prime**	**Positive Response (%)**
**HC (*n* = 53)**	**Masked**	**Control**	44.19
		**Happy**	47.97
		**Sad**	45.26
	**Unmasked**	**Control**	44.49
		**Happy**	49.97
		**Sad**	47.57
**BD (*n* = 14)**	**Masked**	**Control**	54.39
		**Happy**	60.56
		**Sad**	55.96
	**Unmasked**	**Control**	55.38
		**Happy**	63.61
		**Sad**	55.70
**DEP (*n* = 17)**	**Masked**	**Control**	43.02
		**Happy**	40.76
		**Sad**	36.78
	**Unmasked**	**Control**	40.87
		**Happy**	44.29
		**Sad**	42.94

**Figure 6:**
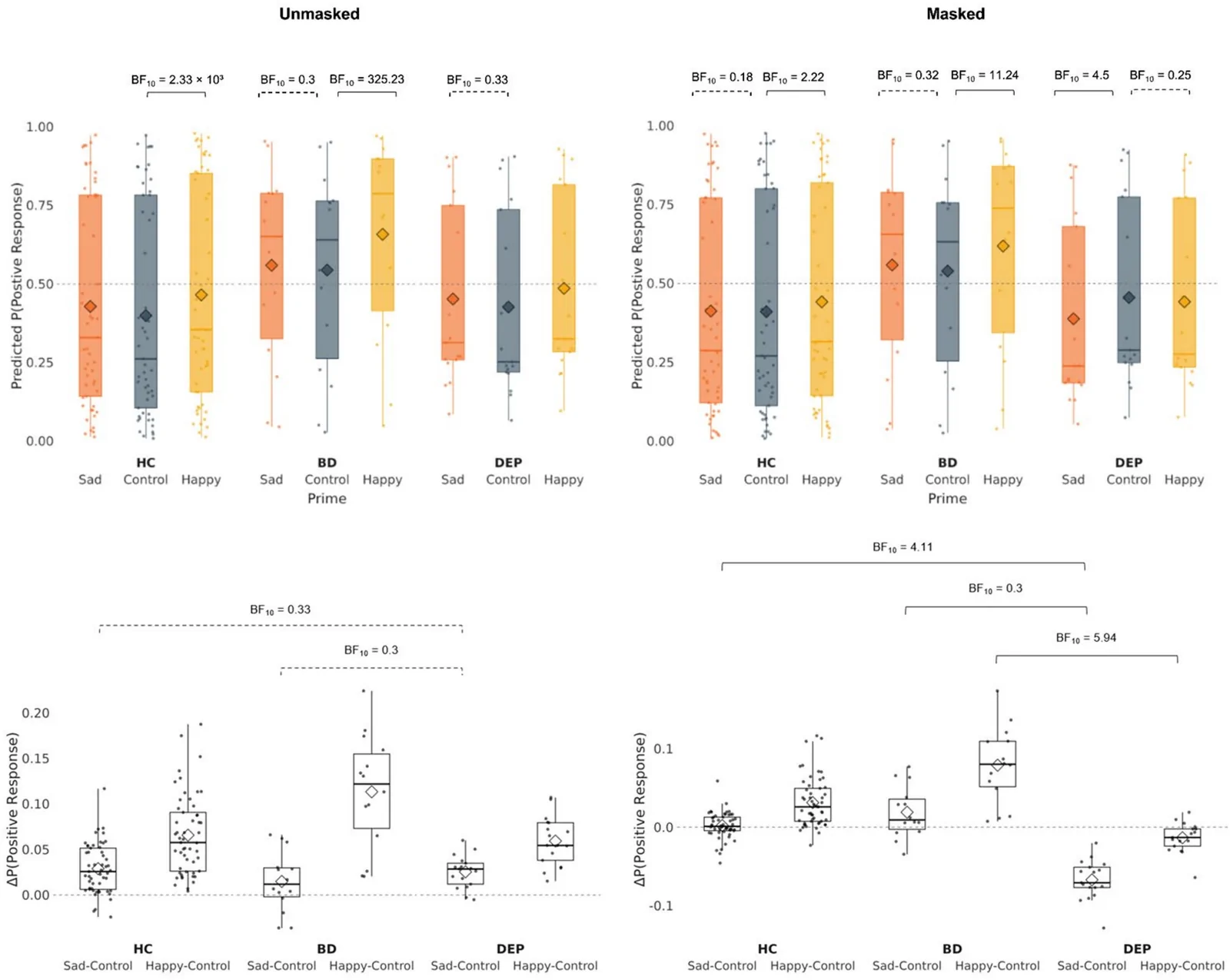
Predicted probability of a positive response to ambiguous morph targets as a function of prime (sad, control, happy) and priming type (masked, unmasked) in HC, BD, and DEP, derived from Bayesian generalized linear mixed-effects models (Bernoulli likelihood). Upper panel: each boxplot displays the distribution of predicted responses across participants. The box represents the interquartile range, horizontal bars indicate the median, and diamonds indicate the mean. Dots represent individual participant estimates. Lower panel: difference in predicted probability of a positive response between happy and control primes (happy-control) and sad and control primes (Sad—Control) for each group, allowing direct comparison of priming effects across groups. BF_10_ values reflect the strength of evidence for the prime effect; brackets indicate evidence in favor of the alternative hypothesis, while dashed brackets indicate evidence in favor of the null hypothesis. Between-group BF_10_ values reflect the strength of evidence for differences in priming effects across groups.

*(3) Priming effects on RTs to ambiguous targets*

*(3a)* In masked trials, we found extremely strong evidence for faster RTs to morphed targets preceded by primes congruent with participants’ responses compared to incongruent primes in HC (β = −0.051, 95% CrI [−0.07,-0.04], BF_10_ = 4.60 × 10^5^) and BD (β = −0.095, 95% CrI [−0.13,-0.06], BF_10_ = 1.28 × 10^4^). In contrast, we found strong evidence for an absence of effect in DEP (β = −0.006, 95% CrI [−0.04,0.02], BF_10_ = 0.09).

In unmasked trials, we found strong evidence for the absence of an effect on RTs to morph targets preceded by congruent versus incongruent primes in HC (β = −0.008, 95% CrI [−0.02,0.01], BF_10_ = 0.1) and moderate evidence for an absence of effect in DEP (β = −0.02, 95% CrI [−0.05,0.01], BF_10_ = 0.22). However, in BD, we found strong evidence for a priming effect (β = −0.054, 95% CrI [−0.13,-0.06], BF_10_ = 17.54).

*(3b)* Regarding between groups differences, in masked trials, there was extremely strong evidence for a stronger priming effect in BD versus DEP (β = −0.089, 95% CrI [−0.13,-0.05], BF_10_ = 509.26), moderate evidence for BD versus HC (β = 0.044, 95% CrI [0.01,0.08], BF_10_ = 3.22), and HC versus DEP (β = −0.045, 95% CrI [−0.08,-0.01], BF_10_ = 5.75). In unmasked trials, there was moderate evidence for a stronger priming effect in BD versus HC (β = 0.046, 95% CrI [0.01,0.08], BF_10_ = 4.68), no conclusive evidence in BD versus DEP (β = −0.034, 95% CrI [−0.08,0.01], BF_10_ = 0.22), and moderate evidence for an absence of differences in priming effect in HC versus DEP (β = 0.013, 95% CrI [−0.02,0.04], BF_10_ = 0.18).

*(3c)* Comparing masked and unmasked trials, the model revealed extremely strong evidence for stronger congruency effect in masked as compared to unmasked trials in HC (β = 0.044, 95% CrI [0.02,0.07], BF_10_ = 108.76), no conclusive evidence for a difference between priming types in BD (β = −0.041, 95% CrI [−0.00,0.08], BF_10_ = 0.82) and strong evidence for no effect in DEP (β = −0.014, 95% CrI [−0.05,0.02], BF_10_ = 0.16).

Finally, in masked trials, results provided moderate evidence for the absence of a differential congruence effect between positive and negative responses to morphs across groups (all 0.17 < BF_10_ < 0.19). In unmasked trials, we found no conclusive evidence for a difference in BD (β = 0.061, 95% CrI [0.00,0.12], BF_10_ = 1.64, ROPE = 4.72%) and DEP (β = 0.053 95% CrI [0.00,0.10], BF_10_ = 1.23, ROPE = 6.69%), but moderate evidence for a stronger priming effects for positive than negative responses in HC (β = 0.038, 95% CrI [0.01,0.07], BF_10_ = 3.6). See Supplementary Fig. S10 for model diagnostics, Tables S20–S24, and in Fig. 7 for results across groups, and Table 1 for mean RTs and SD to morphs by group, prime congruence, and priming type.

**Figure 7:**
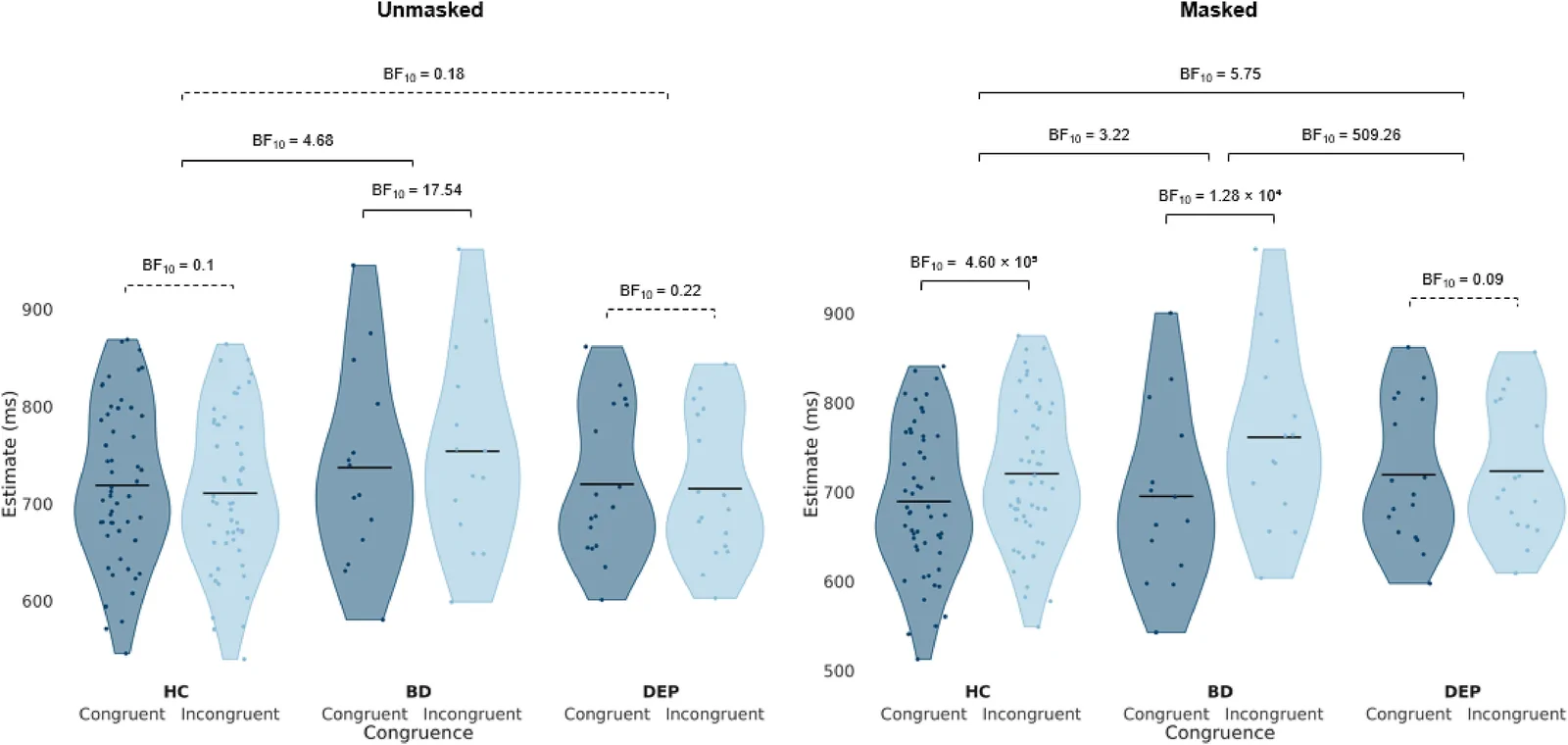
Posterior mean estimates of RTs to ambiguous morph targets as a function of prime congruence (congruent, incongruent) and priming type (masked, unmasked) in HC, BD patients, and DEP patients, derived from Bayesian generalized linear mixed-effects models with log-normal likelihood. Each dot represents the estimated posterior mean RT for one participant, and the horizontal bar indicates the group mean. Violin plots display the distribution and density of estimated RTs across participants. BF_10_ values reflect the strength of evidence for the congruence effect; brackets indicate evidence in favor of the alternative hypothesis, while dashed brackets indicate evidence in favor of the null hypothesis. Between-group BF_10_ values reflect the strength of evidence for differences in congruence effects across groups.

*(4) Effects of mood and primes on perceptual decision making for ambiguous targets (DDM)*

To explore possible latent cognitive processes involved in emotional ambiguity resolution—such as the initial bias before interpretation (starting point) and the speed of evidence accumulation toward a Positive or Negative decision (drift rate)—we employed DDM.

*(4a) Starting point.* We found very strong evidence for an absence of effect of mood on starting point across groups (β = −0.01, 95% CrI [−0.03, 0.01], BF_10_ = 0.08). Exploratory analyses examined this effect within each group, and the model revealed moderate evidence for an absence of effect in HC (β = −0.02, 95% CrI [−0.05, 0.02], BF_10_ = 0.17, and strong evidence no effect in BD (β = −0.015, 95% CrI [−0.06, 0.03], BF_10_ = 0.1), and DEP (β = −0.004, 95% CrI [−0.03, 0.04], BF_10_ = 0.07).

*Drift rate.* We found that the data was 2.14 times more likely under the alternative than the null hypothesis (β = 0.016, 95% CrI [0.01,0.23], BF_10_ = 2.14, with 38.59% of the posterior within the ROPE). Exploratory analyses examined this effect within each group, and that the data was 2.62 times more likely under the alternative than the null for HC (β = 0.142, 95% CrI [0.00, 0.28], BF_10_ = 2.62, with 27.42% of the posterior within the ROPE), and no conclusive evidence for an effect of mood on drift rate in BD (β = 0.058, 95% CrI [−0.14, 0.27], BF_10_ = 0.43), and DEP (β = 0.149, 95% CrI [−0.02, 0.32], BF_10_ = 1.33, ROPE = 28.16).

*(4b) Starting point.* In masked trials, happy primes provided very strong evidence for a positive shift in starting point compared to no primes in HC (β = −0.218, 95% CrI [−0.32, −0.11], BF_10_ = 304.66) and BD (β = −0.345, 95% CrI [−0.54, −0.15], BF_10_ = 110.41), and in DEP, 2.5 times more likely (β = −0.196, 95% CrI [−0.38, −0.01], BF_10_ = 2.51, ROPE = 15.21%). Regarding sad primes, in HC and BD, we found strong evidence for a shift in starting point compared to no primes (HC, β = 0.148, 95% CrI [0.04, 0.25], BF₁₀ = 10.46; BD, β = 0.279, 95% CrI [0.10, 0.47], BF_10_ = 27.10), and moderate evidence for no differences in DEP (β = −0.014, 95% CrI [−0.20, 0.16], BF_10_ = 0.29).

In unmasked trials, the model provided no conclusive evidence for a shift in starting point following happy compared to no primes in HC (β = −0.096, 95% CrI [−0.20, 0.01], BF_10_ = 1.20, ROPE = 52.75%). In BD, the data were 2.09 times more likely under the alternative than the null, though not conclusive (β = −0.193, 95% CrI [−0.38, 0.00], BF_10_ = 2.09, ROPE = 17.11%). In DEP, we found moderate evidence for an effect of happy primes compared to no primes (β = −0.213, 95% CrI [−0.39, −0.03], BF_10_ = 4.07). Regarding sad primes compared to no primes, the model provided moderate evidence for an absence of effect in HC (β = −0.050, 95% CrI [−0.15, 0.05], BF_10_ = 0.37) and no conclusive evidence in favor of H_1_ in BD (β = 0.144, 95% CrI [−0.04, 0.33], BF_10_ = 0.95) and DEP (β = −0.098, 95% CrI [−0.28, 0.08], BF_10_ = 0.52).

*Drift rate.* In masked trials, in HC, we found moderate evidence for an absence of effect of happy primes compared to no primes (β = 0.036, 95% CrI [−0.11, 0.19], BF_10_ = 0.37), and no conclusive evidence for sad primes compared to no primes (β = −0.120, 95% CrI [−0.27, 0.03], BF_10_ = 1.23, ROPE = 39.28%). In BD, the model provided moderate evidence for an absence of effect of happy primes compared to no primes (β = 0.009, 95% CrI [−0.25, 0.26], BF_10_ = 0.42), but moderate evidence that masked sad primes decreased drift rate compared to no primes (β = −0.277, 95% CrI [−0.52, −0.02], BF_10_ = 4.31), reflecting increased accumulation of evidence toward the positive response. In DEP, we found no conclusive evidence for happy primes compared to no primes (β = 0.177, 95% CrI [−0.07, 0.43], BF_10_ = 1.04, ROPE = 25.84%), and the data were 1.95 times more likely under the alternative than the null for sad primes compared to no primes, though not conclusive (β = 0.226, 95% CrI [−0.02, 0.48], BF_10_ = 1.95, ROPE = 15.58%).

In unmasked trials, the model provided very strong evidence that happy primes decreased drift rate compared to no primes in HC (β = −0.250, 95% CrI [−0.40, −0.10], BF10 = 58.81), and moderate evidence for sad versus no primes (β = −0.165, 95% CrI [−0.31, −0.02], BF10 = 3.58), reflecting increased accumulation of evidence toward the negative response independently of the prime valence. In BD, we found strong evidence that happy primes decreased drift rate compared to no primes (β = −0.338, 95% CrI [−0.60, −0.08], BF10 = 10.20), while we found no conclusive evidence for sad primes compared to no primes (β = −0.219, 95% CrI [−0.47, 0.03], BF10 = 1.81, ROPE = 16.74%). In DEP, we found no conclusive evidence for an effect of primes on the drift rate across both contrasts (all 0.41 < BF10 < 0.61).

*(4c–d) Starting point.* For between-group comparisons, in masked primes, we found no conclusive evidence for differences in starting point shifts groups (all 0.45 < BF_10_ < 1.82) in happy versus no prime, and sad versus no prime, except moderate evidence that the shift in starting point following sad primes was larger in BD than in DEP (β = 0.294, 95% CrI [0.04, 0.53], BF_10_ = 6.10).

In unmasked trials, we found no conclusive evidence in favor of H₁ for group differences in starting point for happy versus no primes (all 0.42 < BF_10_ < 0.89). For sad primes compared to no primes, the downward shift in starting point was larger in BD than in both HC and DEP, though neither comparison reached conclusive evidence (HC versus BD: β = −0.194, 95% CrI [−0.39, −0.00], BF_10_ = 2.97, ROPE = 16.82%; BD versus DEP: β = 0.241, 95% CrI [−0.01, 0.49], BF_10_ = 2.39, ROPE = 13.08%), while we found moderate evidence for an absence of difference between HC and DEP (β = 0.047, 95% CrI [−0.14, 0.24], BF_10_ = 0.49).

*Drift rate.* In masked trials, we found no conclusive evidence in favor of H₁ for group differences in drift rate for happy versus no primes (all 0.61 < BF_10_ < 1.05), nor for differences in drift rate for sad versus control primes between HC and BD (β = 0.157, 95% CrI [−0.10, 0.42], BF_10_ = 1.20, ROPE = 30.5%), but strong evidence that the effect of sad primes on drift rate differed between HC and DEP (β = −0.346, 95% CrI [−0.60, −0.09], BF_10_ = 20.44), and very strong evidence for the same difference between BD and DEP (β = −0.503, 95% CrI [−0.85, −0.17], BF_10_ = 39.26).

In unmasked trials, we found moderate evidence for an absence of group differences across all contrasts and group comparisons (0.63 < BF_10_ < 1.18).

*(4e) Starting point.* Comparing masked and unmasked trials, the shift in starting point was slightly larger in masked than unmasked trials in HC for happy versus no primes, with the data being 1.79 times more likely under the alternative than the null, though not conclusive (β = −0.122, 95% CrI [−0.25, 0.01], BF_10_ = 1.79, ROPE = 37.26%). We found no conclusive evidence for block differences in BD (β = −0.152, 95% CrI [−0.38, 0.09], BF_10_ = 0.93) and DEP (β = 0.016, 95% CrI [−0.21, 0.24], BF_10_ = 0.40). Regarding sad primes compared to no primes, we found strong evidence that the downward shift in starting point following sad primes was larger in masked than unmasked trials in HC (β = 0.198, 95% CrI [0.06, 0.33], BF_10_ = 29.02), while we found no conclusive evidence for block differences in both BD (β = 0.136, 95% CrI [−0.09, 0.35], BF_10_ = 0.81) and DEP (β = 0.084, 95% CrI [−0.13, 0.31], BF_10_ = 0.52). See Supplementary Fig. S11 for model diagnostics and Figs 8 and 9 for results across groups.

**Figure 8:**
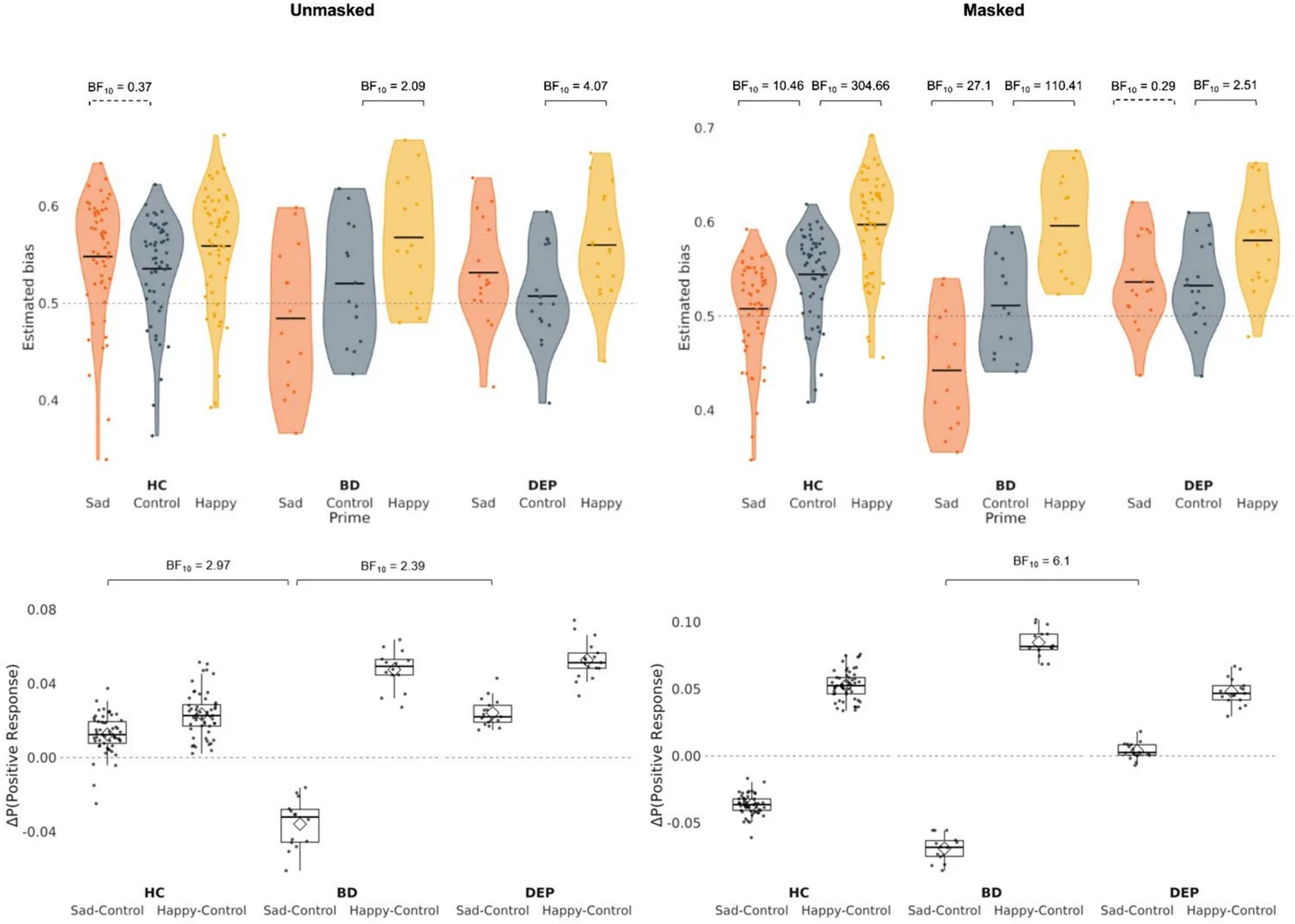
Estimated starting point bias for ambiguous morph targets as a function of prime (sad, control, happy) and priming type (masked, unmasked) in HC, BD patients, and DEP patients, derived from Bayesian hierarchical DDM. Upper panel: Each violin plot displays the distribution and density of individual starting point estimates across participants. Horizontal bars indicate the group mean. A dashed horizontal line indicates the unbiased starting point (0.5). Lower panel: Difference in estimated starting point between happy and control primes (happy-control) and sad and control primes (sad-control) for each group, allowing direct comparison of prime-induced pre-decisional biases across groups. BF_10_ values reflect the strength of evidence for the prime effect; brackets indicate evidence in favor of the alternative hypothesis, while dashed brackets indicate evidence in favor of the null hypothesis. Between-group BF_10_ values reflect the strength of evidence for differences in starting point shifts across groups.

**Figure 9:**
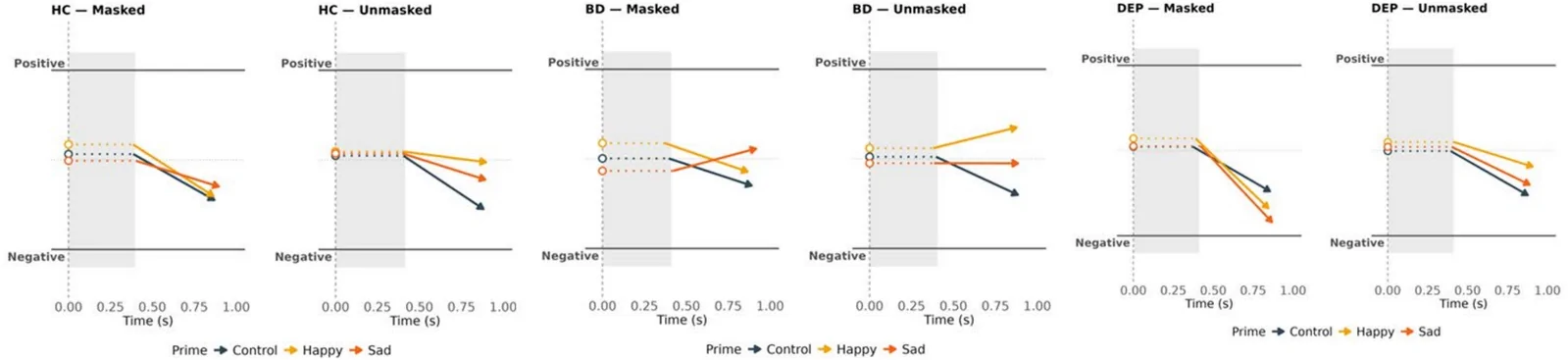
Predicted response trajectories from the DDM for ambiguous morph targets as a function of prime (control, happy, sad) and priming type (masked, unmasked) in HC, BD, and DEP. Each panel displays the predicted decision trajectories toward a positive or negative response over time, for each prime type. Note that these represent drift-rate implied average trajectories, not actual noisy wiener-like trajectories assumed by the DDM. The shaded area indicates the non-decision time. Lines represent the predicted trajectories for control, happy, and sad primes.

*Drift rate.* Comparing masked and unmasked trials, regarding the effect of happy primes compared to no primes, we found very strong evidence that the decrease in drift rate was larger in unmasked than masked trials in HC (β = 0.286, 95% CrI [0.11, 0.46], BF_10_ = 59.40), and moderate evidence for the same pattern in BD (β = 0.346, 95% CrI [0.06, 0.63], BF_10_ = 8.57), and DEP (β = 0.298, 95% CrI [0.01, 0.59], BF_10_ = 4.16). Regarding sad primes compared to no primes, we found no conclusive evidence for block differences in HC (β = 0.045, 95% CrI [−0.13, 0.22], BF_10_ = 0.51) and BD (β = −0.058, 95% CrI [−0.34, 0.24], BF_10_ = 0.57), however, in DEP, the shift in drift rate following sad primes was larger in masked than unmasked trials, with the data being 2.71 times more likely under the alternative than the null, though not conclusive (β = 0.267, 95% CrI [−0.02, 0.55], BF_10_ = 2.71, ROPE = 12.27%). See Fig. 9, Supplementary Tables S25–S30 for DDM results across groups.

### Visibility task

Masked prime detection was above chance in all groups (HC, 78.24% correct, *d′* = 2.12, 95%, CrI [1.75, 2.44], BF_10_ = 1.41 × 10^14^; BD, 65.06% correct, *d′* = 1.20, 95% CrI [0.19, 1.91], BF_10_ = 4.77; DEP, 75.11% correct, *d′* = 1.64, 95% CrI [0.89, 2.19], BF_10_ = 576.47). Happy-sad prime discrimination was above chance in HC (70.62% correct, *d′* = 1.23, 95% CrI [0.87, 1.52], BF_10_ = 2.59 × 10^7^) and DEP (65.70% correct, *d′* = 1.04, 95% CrI [0.2, 1.66], BF_10_ = 5.41), with inconclusive evidence in BD (58.70% correct, *d′* = 0.47, 95% CrI [−0.07, 0.86], BF_10_ = 1.32).

Between groups, no comparison provided conclusive evidence for a difference in prime discriminability. Detection was lower in BD than HC, with the data being 2.59 times more likely under the alternative than the null, though not conclusive (*Δd*′ = −0.92, 95% CrI [−1.56, −0.03], BF_10_ = 2.59). We found no conclusive evidence for differences between BD and DEP (*Δd′* = −0.45, 95% CrI [−1.26, 0.50], BF_10_ = 0.46), nor between HC and DEP (*Δd′* = 0.47, 95% CrI [−0.24, 1.04], BF_10_ = 0.60). Similarly, for happy-sad discrimination the data were 2.35 times more likely under the alternative than the null for BD versus HC, though not conclusive (*Δd′* = −0.76, 95% CrI [−1.31, −0.03], BF_10_ = 2.35), with no conclusive evidence for BD versus DEP (*Δd′* = −0.57, 95% CrI [−1.27, 0.35], BF_10_ = 0.64), and moderate evidence for an absence of difference between HC and DEP (*Δd′* = 0.19, 95% CrI [−0.44, 0.79], BF_10_ = 0.32).

Finally, happy-sad prime discriminability was not associated with the magnitude of priming effects on RTs. For unambiguous targets, we found no conclusive evidence for a relationship in BD (*ρ* = −0.04, 95% CrI [−0.47, 0.41], BF_10_ = 0.56) or DEP (*ρ* = −0.05, 95% CrI [−0.45, 0.36], BF_10_ = 0.52), and moderate evidence for an absence of relationship in HC (ρ = 0.04, 95% CrI [−0.22, 0.29], BF_10_ = 0.32). For ambiguous morph targets, evidence was inconclusive in all groups (HC, ρ = 0.10, 95% CrI [−0.15, 0.35], BF_10_ = 0.43; BD, ρ = −0.21, 95% CrI [−0.60, 0.25], BF_10_ = 0.85; DEP, ρ = 0.23, 95% CrI [−0.21, 0.58], BF_10_ = 0.93). See Supplementary Fig. S12.

## Discussion

In this study investigating the influence of mood (endogenous predictions) and affective primes (exogenous predictions) on emotional perception, we found that emotionally congruent primes facilitated the recognition of emotional targets, with differential effects across groups and priming type (masked, unmasked), while mood did not influence ambiguous emotional categorization in any group, though anecdotal evidence for a subtle influence on drift rate was observed, specifically in HC.

First, contrary to our expectations and previous results reporting mood-congruent biases in ambiguous perception (*e.g.*  Beevers et al. 2009, Qiao-Tasserit et al. 2017, Trilla et al. 2021), we did not find conclusive evidence for an effect of mood on morph categorization, whether across or within groups. Note that the Bayesian analyses were non-conclusive rather than strongly in favor of the null hypothesis (*i.e.* no effect), and the complexity of the model may have limited statistical power. Another possible interpretation relates to the sensitivity of our paradigm. Previous studies showing mood-congruent biases in ambiguous interpretation used morphing continuum ranging from 0% to 100% (Beevers et al. 2009, Qiao-Tasserit et al. 2017), measuring for example the point of subjective equality (*i.e.* the point at which participants are equally likely to categorize the face as positive or negative; Qiao-Tasserit et al. 2017). In contrast, our paradigm relied on fixed 50%–50% morphs with binary decisions, which may have lacked the sensitivity to detect the influence of long-lasting, more diffuse mood states—whose effects on perception may be too small to shift binary responses—while remaining sensitive to exogenous affective primes. Moreover, contrary to our predictions, DDM showed strong evidence for no effect of mood on starting point across groups, suggesting that mood did not induce pre-decisional affective biases favoring a positive or negative response before stimulus onset. This contrasts with White et al. (2018), who reported mood-congruent shifts in starting point following brief mood inductions, though it is worth noting that such transient induced affective states may act more similarly to exogenous predictions than endogenous, longer-lasting mood states measured here, potentially explaining why their effect on early decision dynamics were more easily detectable. However, the data were approximately twice as likely under the alternative than the null hypothesis for an effect of mood on drift rate, across groups and specifically in HC, suggesting that long-lasting mood states may still subtly influence the weighting of emotional features during ambiguous face perception (*i.e.* the attention given to positive or negative features of the morph faces), without being sufficient to shift the final categorical response.

Affective primes influenced the categorization of ambiguous faces in a group- and valence- specific manner. HC and BD rated morphs more positively following happy primes (masked or unmasked), while sad primes had no effect in neither groups in masked trials. In contrast, DEP showed more negative ratings following masked sad primes, with stronger evidence than HC and BD, while happy primes had no effects. The effects of sad primes were greater in masked than unmasked trials in DEP. Although discrimination rate was above chance in the visibility task, a putative explanation is that this bias operates preferentially under unconscious conditions. Such a result deserves to be replicated in a strict conscious versus unconscious paradigm. These results align with previous masked priming findings in depression (Suslow et al. 2010, Klug et al. 2024) and the well-established negativity bias (Gotlib and Joormann 2010). Such negative biases may reinforce negative interpretations of emotional ambiguity, thereby contributing to the persistence of depressive episodes (Penton-Voak et al. 2017).

Priming effects on RTs to ambiguous target faces also differed across groups. HC were faster at categorizing morphs as positive or negative after masked positive or negative primes, respectively, while strong evidence for no facilitation was observed with unmasked primes. These results suggest that masked primes facilitate emotional categorization and ambiguity resolution more than unmasked primes. Although discrimination rate was above chance in the visibility task, a possible explanation is that masked primes may trigger more automatic processing compared to visible primes, thereby facilitating ambiguity resolution more strongly. Such results deserve to be replicated in a paradigm with stricter control of prime awareness. In contrast, BD patients exhibited priming effects regardless of priming type, with stronger facilitation than both HC and DEP in masked trials, and stronger than HC in unmasked trials, which may indicate reduced top-down control and greater reliance on external input—consistent with impaired emotion regulation in BD (Townsend and Altshuler 2012, Phillips and Swartz 2014). This pattern is also consistent with recent evidence showing higher brain plasticity in BD than MDD, associated with greater likelihood of mental state transitions (Viglione et al. 2025), which may contribute to mood instability, emotional hyperreactivity, and heightened sensitivity to external affective cues (*e.g.* affective primes). From a predictive coding perspective, our results may reflect low-precision mood priors, leading to an over-reliance on external affective cues to guide perception. Such dysregulation could contribute to mood instability by amplifying emotional responses to ambiguous or contextually irrelevant stimuli (Henry and Berkovitch 2024). DEP patients overall showed strong evidence for no priming effects, consistent with emotional blunting (Christensen et al. 2022, Ma et al. 2021) and reduced responsiveness to both positive and negative stimuli found in depression (*i.e.* emotion context insensitivity; Rottenberg and Hindash 2015). From a predictive coding perspective, mood may act as an overly strong prior, resisting updating from external cues and reducing perceptual flexibility.

These group differences are unlikely to be driven by differences in prime visibility. HC and DEP showed comparable happy-sad discriminability, with moderate evidence for no difference between them, yet contrasting priming patterns: facilitation in HC on masked trials, and evidence for no priming in DEP in either condition. Conversely, BD showed the lowest discriminability of the three groups—with inconclusive evidence that it exceeded chance—alongside strong priming effects, stronger than both HC and DEP on masked trials, and then HC on unmasked trials, the opposite of what a visibility-driven account would predict. The stronger masked than unmasked priming observed in HC is similarly difficult to attribute to prime visibility, since unmasked primes were fully visible by design; a visibility account would predict the reverse. At the individual level, we found no evidence that the magnitude of priming on morphs varied with happy-sad discriminability in any group. These comparisons were nonetheless inconclusive rather than in favour of the null, and replication in larger samples, with stricter control of prime awareness, would be needed to determine whether prime visibility contributes to these group differences.

DDM further informed these group- and valence-specific effects. In masked trials, happy and sad primes shifted the starting point towards positive and negative responses, respectively, in HC and BD, that is, they biased participants’ initial expectations toward a positive or negative response before the target appeared, consistent with a PP account in which affective cues shape prior expectations. The downward shift in starting point following masked primes was larger in BD than HC and DEP, consistent with stronger reliance on external affective cues. Contrary to our expectations and categorization results, in DEP the starting point was slightly shifted towards positive responses after happy primes, and there was evidence for no effects following sad primes. One possible interpretation is that the negativity bias in DEP does not operate at the level of precisional decisional expectations (*i.e.* starting point), but rather at a later stage of evidence accumulation, possibly reflected by the drift rate (though the evidence here was not sufficient to draw conclusion; data were only approximately twice as likely under the alternative than the null hypothesis for an effect of sad primes on drift rate in DEP). In contrast, in unmasked trials, we found no conclusive evidence for starting point shifts induced by affective primes across groups, except a moderate shift towards positive interpretations in DEP following unmasked happy primes, a finding that is difficult to interpret within the current framework, and require replications in future studies. Similarly, priming effects on drift rates across groups and priming types were unexpected and their interpretation remains unclear at this stage.

Regarding RTs to unambiguous happy and sad targets, consistent with prior research (Neumann and Lozo 2012, Rohr et al. 2012, Wentura and Rohr 2018), emotionally congruent prime faces facilitated the recognition of target emotions across all groups in masked trials, and only in HC and BD in unmasked trials, with no differences between groups. This supports PP accounts, suggesting that primes pre-activate specific emotion categories, thereby facilitating emotional recognition. In DEP, this effect was limited to masked primes, with evidence for no effect in unmasked trials. Together with the absence of priming effects on categorization and RTs to ambiguous targets in unmasked trials, this suggests a generalized reduced sensitivity external affective cues presented as unmasked primes in DEP, consistent with the context insensitivity hypothesis (Rottenberg and Hindash 2015) and the idea of rigid mood priors resisting updating from external affective inputs and reducing perceptual flexibility (Kube et al. 2020, Bottemanne et al. 2022, 2023, Ramos-Grille et al. 2022).

A major limitation is the use of self-reported diagnosis without standardized assessment; no data were collected on symptom severity, episode type, illness duration, and treatments. The online format with small clinical samples further limits generalizability, and the *a priori* power calculation did not account for the complexity of the models reported here. Future studies should recruit larger, clinically assessed samples in controlled settings. Our mood index was also derived from two self-report scales only (PANAS and MAThyS), which provide a global measure of current affect but cannot dissociate closely related constructs such as behavioural activation/inhibition (*e.g.* motivation, reward sensitivity, activity levels). Including scales designed to capture these constructs—as well as measures of emotional blunting, which may be particularly relevant to the reduced sensitivity to affective primes observed in DEP—would allow a finer characterisation of which affective dimensions drive predictive biases in emotional perception. Illness severity and chronicity—such as number of hospitalizations, suicide attempts, and illness duration—may amplify affective predictive biases, with more severe and chronic illness expected to be associated with more trait-level biases, even between mood episodes. In BD, mood scores were comparable to HC, suggesting that they were likely euthymic. The stronger priming effects observed regardless of priming type (*i.e.* masked or unmasked) may thus reflect trait-level emotional hyper-reactivity, well-established in BD even during euthymia (Henry et al. 2008b, M’bailara et al. 2009). During mood episodes, these biases may become more valence-specific, with stronger negative predictions expected during depressive episodes, and stronger positive predictions during manic states (García-Blanco et al. 2013, Mason et al. 2017, Ivanova et al. 2025). Even during depressive episodes, BD patients may still show more sensitivity to external affective cues than DEP. Consistently, from a clinical perspective, some patients with BD usually have more ‘reactive’ depression compared to unipolar patients (Henry et al. 2012). Moreover, previous studies suggest that BD patients have higher plasticity levels and greater likelihood of transitioning between mental states regardless of depressive symptom severity compared to unipolar patients (Viglione et al. 2025). In DEP, lower mood scores suggest more negative affect at the time of the task compared to HC. However, it is not possible to differentiate trait-level affective biases, influenced by illness history, from state-dependent effects linked to a current depressive episode. Interestingly, Münkler et al. (2015) demonstrated that biases in ambiguous emotional perception in DEP likely reflect current clinical states rather than stable trait-level characteristics. Taken together with our results, this suggests that affective predictive biases observed in DEP may be primarily state-dependent, in contrast to BD, where emotional hyperreactivity and greater sensitivity to external affective cues may persist as trait-level characteristics even during euthymia. Finally, pharmacological treatments add further complexity in the interpretation of the results, as while antidepressants may alleviate negative emotional biases (Harmer and Cowen 2013, Y. Ma 2015, Martens et al. 2025), they are also associated with emotional blunting as a side effect (Goodwin et al. 2017, Jawad et al. 2023, Ma et al. 2021), which may also explain the reduced sensitivity to external cues observed in DEP. Future studies should investigate whether treatments specifically targeting affective predictive biases—such as ketamine (Sumner et al. 2020, Bottemanne et al. 2023, Harvey et al. 2023) or psilocybin (Stroud et al. 2018, Doss et al. 2021) can restore perceptual flexibility and sensitivity to external affective cues in mood disorders. More broadly, the group-level patterns reported here—including the trait- and state-level interpretations proposed above—should be regarded as hypothesis-generating and require confirmation in clinically assessed samples before being generalized to BD and MDD populations.

## Conclusion

In sum, our findings suggest different predictive biases in emotional perception in individuals with and without declared mood disorders. While mood (endogenous predictions) did not influence ambiguous face categorization in any group, it showed a subtle influence on the weighting of emotional features during ambiguous face perception (*i.e.* the attention given to positive or negative features of the morph faces), particularly in HC. Affective primes (exogenous predictions) influenced ambiguous emotional perception in a group-, valence- and priming-type-dependent manner: HC showed priming effects primarily for masked primes, BD showed robust effects regardless of priming type, with stronger facilitation than HC and DEP, suggesting greater reliance on external affective cues, and DEP showed overall reduced sensitivity to affective primes, with the exception of a selective negativity interpretation bias induced by masked sad primes. DDM further revealed dissociation between affective primes and mood: exogenous predictions induced pre-decisional biases towards positive or negative responses to ambiguous morphs—that is, before the morph was even perceived—while mood subtly influenced the weighting of emotional features during morph perception. Taken together, these results provide preliminary evidence that biased affective predictions—whether unstable or overly strong—may underlie mood disorders and represent promising targets for therapeutic interventions aiming to restore adaptive emotional inference, such as cognitive-behavioural, mindfulness-based, or psychedelic-assisted therapies.

## Supplementary Material

Supplementary_materials_niag072

## Data Availability

The data that support the findings of this study are available on request from the corresponding author. The data are not publicly available due to privacy or ethical restrictions.

## Permission to reproduce material from other sources

We used publicly available FACES objects to illustrate our research methodology (Fig. 1), and had permission to adapt Fig. 2 from Lacroix et al. (2021) to our paradigm.
