# Supplementary material for: Predictive biases in emotional perception: differential influence of mood and affective primes in individuals with and without mood disorders
Source: Neurosci Conscious. 2026 Oct 31;2026(1):niag072. doi: 10.1093/nc/niag072 (PMC13723272; doi:10.1093/nc/niag072)
Supplement: Supplementary file 1 [file supplementary_materials_niag072.docx]

**Supplementary Materials**

**Predictive Biases in Emotional Perception: Differential Influence of Mood and Affective Primes in Individuals with and without Mood Disorders**

Alexane Leclerc, Ladislas Nalborczyk, Louise Kauffmann, Yann Leprince, Josselin Houenou, Lucie Berkovitch, Pauline Favre

**Table of content**

[Supplementary Methods](#_Toc205064291)	4

[Supplementary Methods S1. Sample size calculation](#_Toc510973584)	4

[Supplementary Methods S2. Participant’s recruitment and patients pre-screening](#_Toc822633683)	5

[Supplementary Methods S3. Exclusion criteria](#_Toc1174568874)	6

[Supplementary Methods S4. Stimuli and experimental design](#_Toc329726430)	7

[Supplementary Methods S5. PCA pipeline for deriving the composite mood score](#_Toc1549867939)	8

[Supplementary Methods S6. Bayesian Linear mixed-effects models analyses](#_Toc1557996440)	9

[Supplementary Methods S7. Drift Diffusion Modeling	1](#_Toc297723054)3

[Supplementary Methods S8. Prime visibility task	1](#_Toc1926622418)5

[Supplementary Methods S9. Correlation between Reactivity Index (PC3) and Reaction Time Priming Effects	1](#_Toc336692965)6

[Supplementary Tables	1](#_Toc451637621)7

[Supplementary Table S1. Demographic characteristics for HC, BD, and DEP.	1](#_Toc1615932604)7

[Supplementary Table S2. Summary of hypotheses and results.	1](#_Toc1027683778)8

[Supplementary Table S3. Questionnaire scores for HC, BD, and DEP.](#_Toc2028031464)	20

[Supplementary Table S4. Feature contribution in PC1	2](#_Toc2107861505)2

[Supplementary Table S5. Feature contribution in PC2	2](#_Toc57666898)4

[Supplementary Table S6. Feature contribution in PC3	2](#_Toc2117159363)6

[Supplementary Table S7. Feature contribution in PC4	2](#_Toc1356449147)8

[Supplementary Table S8. Proportion of correct responses per Priming Type, Group, Congruence, and Target](#_Toc1280128991)	30

[Supplementary Table S9. Model comparison for reaction times to unambiguous targets	32](#_Toc46182486)

[Supplementary Table S10. Bayesian estimates of priming effects on reaction times (Congruent – Incongruent) for unambiguous targets across groups and priming type	33](#_Toc943168777)

[Supplementary Table S11. Bayesian estimates of priming effects on reaction times (Congruent – Incongruent) for unambiguous targets between groups and across priming type	34](#_Toc2069640851)

[Supplementary Table S12. Bayesian estimates of priming effects on reaction times (Congruent – Incongruent) for unambiguous targets between priming type and across group	35](#_Toc427574854)

[Supplementary Table S13. Bayesian estimates of priming effects on reaction times (Congruent – Incongruent) for unambiguous targets between target valence across groups and priming type	36](#_Toc1497534347)

[Supplementary Table S14. Model comparison for responses to ambiguous targets	37](#_Toc366564214)

[Supplementary Table S15. Bayesian estimates for the effects of mood (as indexed by PC1) on morph responses	38](#_Toc907011234)

[Supplementary Table S16. Bayesian estimates for the effects of mood (as indexed by PC1) on morph responses across groups	39](#_Toc1492285325)

[Supplementary Table S17. Bayesian estimates for the effects of primes on morph responses across groups and priming type	40](#_Toc974217986)

[Supplementary Table S18. Bayesian estimates for the effects of primes on morph responses between groups across priming type	41](#_Toc591595287)

[Supplementary Table S19. Bayesian estimates for the effects of primes on morph responses between priming type across groups	42](#_Toc548208438)

[Supplementary Table S20. Model comparison for reaction times to ambiguous targets	43](#_Toc1055426785)

[Supplementary Table S21. Bayesian estimates of priming effects on reaction times (Congruent – Incongruent) for ambiguous targets across groups and priming type	44](#_Toc136958126)

[Supplementary Table S22. Bayesian estimates of priming effects on reaction times (Congruent – Incongruent) for ambiguous targets between groups and across priming type	45](#_Toc547866709)

[Supplementary Table S23. Bayesian estimates of priming effects on reaction times (Congruent – Incongruent) for ambiguous targets between priming type and across group	46](#_Toc417486537)

[Supplementary Table S24. Bayesian estimates of priming effects on reaction times (Congruent – Incongruent) for ambiguous targets between target valence across groups and priming type	47](#_Toc836879829)

[Supplementary Table S25. Model comparison for drift diffusion models	48](#_Toc588999906)

[Supplementary Table S26. Drift Diffusion Modeling results for the effect of primes on starting point and drift rate across groups and priming type	49](#_Toc819637324)

[Supplementary Table S27. Drift Diffusion Modeling results for the effect of primes on starting point and drift rate between groups across priming type	50](#_Toc450958488)

[Supplementary Table S28. Drift Diffusion Modeling results for the effect of primes on starting point and drift rate between priming type across groups	51](#_Toc2059355852)

[Supplementary Table S29. Drift Diffusion Modeling results for the effect of mood on starting point and drift rate across groups - All groups by primes	52](#_Toc1021896401)

[Supplementary Table S30. Drift Diffusion Modeling results for the effect of mood on starting point and drift rate across groups - All groups all primes	53](#_Toc1409122936)

[Supplementary Results	54](#_Toc153036421)

[Supplementary Results S1. No significant correlation between Reactivity Index (PC3) and Reaction Time Priming Effects	5](#_Toc884150814)4

[Supplementary Figures	55](#_Toc1630068757)

[Supplementary Figures S1. Loadings and group differences on PC2	5](#_Toc528287039)5

[Supplementary Figures S2. Loadings and group differences on PC3	56](#_Toc837857573)

[Supplementary Figures S3. Loadings and group differences on PC4	57](#_Toc765701910)

[Supplementary Figures S4. Correlation between PC3 (Reactivity Index) and Reaction Time Priming Effects on unambiguous targets (happy/sad) preceded by masked primes	58](#_Toc1165275922)

[Supplementary Figures S5. Correlation between PC3 (Reactivity Index) and Reaction Time Priming Effects on unambiguous targets (happy/sad) preceded by unmasked primes	59](#_Toc319400237)

[Supplementary Figures S6. Correlation between PC3 (Reactivity Index) and Reaction Time Priming Effects on ambiguous targets (morphs) preceded by masked primes	60](#_Toc29056687)

[Supplementary Figures S7. Correlation between PC3 (Reactivity Index) and Reaction Time Priming Effects on ambiguous targets (morphs) preceded by unmasked primes	61](#_Toc464837031)

[Supplementary Figures S8. Posterior predictive checks and MCMC diagnostics for the Bayesian multilevel model on RTs to unambiguous targets	62](#_Toc363357943)

[Supplementary Figures S9. Posterior predictive checks and MCMC diagnostics for the Bayesian multilevel model on responses to ambiguous targets	63](#_Toc1958739172)

[Supplementary Figures S10. Posterior predictive checks and MCMC diagnostics for the Bayesian multilevel model on RTs to ambiguous targets	64](#_Toc1047644803)

[Supplementary Figures S11. Posterior predictive checks and MCMC diagnostics for the drift diffusion model on ambiguous targets	65](#_Toc429757751)

[Supplementary Figures S12. Correlation between d’ happy-sad and RT priming effects in HC, BD and DEP.	66](#_Toc108394166)

[References	67](#_Toc1469586157)

# Supplementary Methods

## Supplementary Methods S1. Sample size calculation

An a priori power analysis based on Fisher’s r-to-z transformation was conducted using an expected correlation of r = 0.4, derived from Qiao-Tasserit et al. (2017), who reported a relationship between depressive symptom severity (Beck Depression Inventory scores) and the categorization of ambiguous emotional facial morphs (happy–angry). Assuming a two-sided Bonferroni-corrected significance level of α = 0.025, 80% power, and a 15% attrition rate due to online and technical issues, the target sample size was set to n = 66. Note that this calculation did not account for the complexity of the models reported in the manuscript.

## Supplementary Methods S2. Participant’s recruitment and patients pre-screening

French-speaking participants were recruited through Prolific ([www.prolific.co](http://www.prolific.co/)) and paid £8 an hour for their participation.

Clinical groups (BD and DEP):

Participants with mood disorders were pre-screened using Prolific platform-wide filters, based on self-reported answers to the following:

(1) “Mental Health Diagnosis”: Yes
(2) “Mental health/illness/condition - ongoing”: Yes
(3) “Mental Health Diagnosis Detail”: Bipolar Disorder (for the BD group); Depression (for the DEP group)

We recruited all French-speaking participants with a diagnosis of BD available on the Prolific platform at the time of inclusion.

## Supplementary Methods S3. Exclusion criteria

**Control of prime presentation durations.**

A quality check was performed on the effective presentation duration of primes for each participant. For each individual, we computed the proportion of trials in which prime durations fell outside the predefined temporal window (< 20 ms or > 45 ms). Participants with more than 15% of trials outside this window were excluded from all analyses (N = 13). This window was defined based on the nominal prime duration (~33 ms, corresponding to 2 frames at 60 Hz). Durations shorter than 20 ms most likely correspond to a single frame (~17 ms), which is considered too brief and unstable, whereas durations longer than 45 ms approach three frames (~50 ms), increasing the risk that the prime becomes consciously perceived. In addition, all trials with prime durations < 25 ms or > 40 ms were excluded from the analyses, regardless of participant exclusion, as a conservative quality-control step to ensure that every analysed trial unambiguously corresponded to a two-frame presentation. This additional trimming represented 2.76% of trials.

Task comprehension was assessed using responses to unambiguous emotional targets (happy and sad faces). Participants showing aberrant performance on these trials were excluded. Exclusion was based on performance values falling below 2 standard deviations from the mean (N = 5).

**Detection of aberrant reaction times at the participant level.**

RTs were examined separately for happy and sad targets for each group. Participants with mean RTs falling below or above 2 standard deviations from the group mean were excluded (N = 5). A conservative criterion was adopted given the online nature of the experiment.

**Exclusion of trials with aberrant reaction times.**

At the trial level, RTs exceeding 3000 ms were excluded, corresponding to the theoretical upper limit of the task and reflecting technical issues related to online lag. In addition, RTs falling below or above 3 standard deviations from the mean were excluded. These thresholds were computed separately for each target type and each group, in order to remove excessively fast or slow responses likely reflecting attentional lapses or network-related delays. These reaction-time criteria removed 1.65% of trials.

## Supplementary Methods S4. Stimuli and experimental design

Stimuli were created using happy and sad facial expressions of 24 individuals (12 females) from the FACES database [(Ebner, Reidiger, & Lindenberger, 2010)](https://www.zotero.org/google-docs/?Kb8xF1). For each individual, we generated a morph by equally blending happy and sad expressions using Abrosoft Fantamorph Deluxe 5.0 (2011, [https://www.fantamorph.com](https://www.fantamorph.com/)). This resulted in a total of 72 stimuli (24 happy, 24 sad, 24 morphs). We equalized the luminance of the images in greyscale and applied an oval occluding hair and clothing. A mask with the mean amplitude spectrum frequency was created to avoid retinal persistence between the prime and target.

The experiment was designed on PsychoPy [(Peirce et al., 2019)](https://www.zotero.org/google-docs/?fN8uUB) and ran online via Pavlovia ([https://pavlovia.org](https://pavlovia.org/)). Participants were presented with emotional target faces (happy, sad, or morphed) while being primed with a masked or unmasked happy, sad, morph prime face, or a control condition (blank oval). Their task was to rate as quickly as possible the valence of the emotion perceived on the target (Positive or Negative). Prime-target pairings differed in identity, matched gender, and avoided consecutive repetition. The content of a trial is depicted in *Figure 1*. In total, there were 864 randomized trials, evenly split across conditions. The code used to build the experiment can be found on GitHub (<https://github.com/alexvnne/Leclerc_et_al_2025>).

## Supplementary Methods S5. PCA pipeline for deriving the composite mood score

To investigate the effect of mood on the categorization of morphed targets, we first extracted a unidimensional measure of mood from the 47 items of the two mood questionnaires using Principal Component Analysis (PCA). All items were standardized, and those with null variance were discarded. We then computed a covariance matrix to assess correlations among the items, and Principal Components (PCs) were extracted as linear combinations of the original items. Significance of PCs was computed with a permutation-based non-parametric test with 10,000 iterations. At each iteration, the order of responses was randomly shuffled before re-running the PCA, generating a null distribution [(Ji et al., 2021; Lee et al., 2024; Moujaes et al., 2024](https://www.zotero.org/google-docs/?VBCkjW)).

Group differences in each significant PC were assessed using a linear model with group (HC, BD, DEP) as a categorical predictor.

## Supplementary Methods S6. Bayesian Linear mixed-effects models analyses

*1. Unambiguous Emotional Targets (Happy/ Sad):*

RTs to unambiguous targets (happy and sad combined) were analyzed using a Bayesian hierarchical generalized linear mixed-effects model with a log-normal likelihood implemented in the *brms* package (Bürkner, 2017). Congruence (Congruent, Incongruent), Priming Type (Masked, Unmasked), Group (HC, BD, DEP), and their interactions were included as fixed effects, and Target Valence (Happy, Sad) as a covariate. Random effects included by-participant intercepts and slopes for Priming Type × Congruence, as well as by-target intercepts. Models were estimated with 8 Monte Carlo Markov Chains (MCMC) with 6,000 iterations each, including 1,000 warm-up, resulting in a total of 40,000 posterior samples for statistical inference. Priors and the model were specified as folltheows:

**Priors**

*priors <- c(*

*prior(normal(log(0.6), 0.15), class = "Intercept"),*

*prior(normal(0, 0.1), class = "b"),*

*prior(exponential(10), class = "sd"),*

*prior(exponential(10), class = "sigma")*

*)*

**Formula**

*RT ~ 1 + Congruence × PrimingType × Group + TargetValence +*

*( 1 + Congruence × PrimingType | Participant) +*

*(1 | Target)*

*2. Ambiguous Emotional Targets (Morphs)*

*2.1 Categorization of Morphs (% of positive responses)*

To investigate the effects of *(a)* Mood and *(b)* Emotional Primes on responses to ambiguous morph targets (Positive, Negative), we fitted a Bayesian hierarchical generalized linear mixed-effects model with a Bernoulli likelihood and logit link. The emotional expression of the Prime (Happy, Sad, Control), Priming Type (Masked, Unmasked), Group (HC, BD, DEP), Mood (PC1), and their interactions were included as fixed effects. Random effects included by-participant intercepts and slopes for Prime × Priming Type, as well as by-target intercepts. Models were estimated with 8 MCMC chains (6,000 iterations; 1,000 warm-up), resulting in a total of 40,000 posterior samples. Priors and model were specified as follows:

**Priors**

*priors <- c(*

*prior(normal(0, 0.5), class = "Intercept"),*

*prior(normal(0, 0.5), class = "b"),*

*prior(exponential(1), class = "sd")*

*)*

**Formula**

*Response ~ 1 + Prime × PrimingType × Group × PC1 +*

*( 1 + Prime × PrimingType | Participant) + (1 | Target)*

*2.2 Reaction Times to Morphs*

We performed a Bayesian hierarchical generalized linear mixed-effects model with a log-normal likelihood to investigate RTs to morphed targets rated as positive or negative preceded by either primes congruent or incongruent with participants’ response. The model included fixed effects for Prime Congruence with Response (Congruent, Incongruent), Priming type (Masked, Unmasked), Group (HC, BD, DEP), and their interactions, and Response Valence (Positive, Negative) was added as a covariate but was not of primary interest. Random effects included by-participant intercepts and slopes for Congruence × Priming Type, as well as by-target intercepts. Models were estimated with 8 MCMC chains (6,000 iterations; 1,000 warm-up), resulting in a total of 40,000 posterior samples. Prior specification was the same as in *(1)* and model was specified as follows:

*RT ~ 1 + Congruence × PrimingType × Group + ResponseValence +*

*( 1 + Congruence × PrimingType | Participant) + (1 | Target)*

The fixed-effects structure of each model was specified a priori from our hypotheses and retained across all analyses. Age and Sex, in contrast, were treated as demographic covariates carrying no associated hypothesis. For each model, we therefore compared the pre-specified specification with a version including Age and Sex as additional fixed effects, using Leave-One-Out Cross-Validation (LOO-CV; Vehtari et al., 2017), which estimates out-of-sample predictive accuracy. The Expected Log Pointwise Predictive Density (ELPD) was used as the primary criterion: higher ELPD indicates better predictive performance. Model weights were computed to quantify the probability that each model provides the best predictions. For the best-fitting model, the mean of the posterior distribution and the Bayes factor (BF_10_) were reported for the effects of interest (as outlined in the research objectives and hypotheses), with the latter providing a measure of relative evidence for the alternative relative to the null hypotheses [(e.g., Wagenmakers, 2007)](https://www.zotero.org/google-docs/?BFw3iZ). Following [Wagenmakers et al. (2018](https://www.zotero.org/google-docs/?FMUYyZ)), we interpret BF_10_ values continuously but also provide thresholds for easier interpretation: anecdotal (BF_10_ 1–3), moderate (3–10), strong (10–30), very strong (30–100), and extreme evidence (> 100). Conversely, values below 1/3 provide moderate evidence in favor of the null hypothesis. BF10 values between 1/3 and 3 are considered inconclusive, indicating no substantial support for either hypothesis. We also report the region of practical equivalence (ROPE) to quantify the proportion of the posterior distribution falling within a range of negligible effect size, complementing the interpretation of Bayes Factors when evidence is ambiguous. ROPE boundaries were defined separately for each model based on the scale of the outcome variable and the distributional family used. For the morph categorization model (Bernoulli), the ROPE was set to [-0.18, 0.18] on the log-odds scale. For RT models (log-normal), the ROPE was set to [-0.016, 0.016] for unambiguous targets and [-0.014, 0.014] for ambiguous targets, corresponding to negligible effects on the log-RT scale. For DDM parameters, ROPE boundaries were set to [-0.1, 0.1] for drift rate, starting point bias, and boundary separation, and to [-0.05, 0.05] for non-decision time, reflecting the smaller scale of this parameter.

Note that multiple comparisons were not corrected for, as the hierarchical structure of our Bayesian mixed-effects models induces partial pooling across participants and items, which inherently regularizes estimates, an integrated approach to the multiple comparisons problem (Gelman et al., 2009).

**Contrasts**

All pairwise contrasts were estimated with the *emmeans* package, following the order of the factor levels (Prime: Control, Happy, Sad; Group: HC, BD, DEP; Priming Type: Masked, Unmasked; Congruence: Congruent, Incongruent; Target Valence: Happy, Sad; Response Valence: Negative, Positive). Contrasts are therefore reported as Control − Happy and Control − Sad for primes, HC − BD, HC − DEP and BD − DEP for groups, Masked − Unmasked for priming type, and Congruent − Incongruent for congruence, Happy − Sad for target valence, and Negative − Positive for response valence.

## Supplementary Methods S7. Drift Diffusion Modeling

DDM models decision-making as accumulation of information or evidence over time until a decision threshold is reached (in our case; resulting in either a “Positive” or “Negative” response). In a two-alternative forced-choice task, each option corresponds to a threshold, or “boundary”, with reaction times measured for each trial and each participant. DDMs estimate several latent cognitive processes: the drift rate (delta) is the speed of information accumulation towards one boundary. A higher drift rate indicates faster evidence accumulation. The threshold distance (alpha) is the amount of information required to make a decision. A higher threshold distance signifies more cautious decision-making. Non-decision time (tau) represents the time spent on processes other than decision-making, such as motor response and stimulus encoding. The starting point (beta) is the initial bias in the decision-making process. A higher starting point represents a starting point biased towards the upper boundary (i.e., responding “Positive”), and vice versa.

All analyses were conducted in R. We fitted a Bayesian multilevel model using the *brms* package [(Bürkner, 2017)](https://www.zotero.org/google-docs/?UzGo76) on morphed targets only, with three categorical predictors - Prime (Control, Happy, Sad), Priming Type (Masked and Unmasked) and Group (HC, BD, DEP) - alongside Mood (PC1 score) as a continuous predictor, including all main effects and their interactions. We specified a random intercept for each participant and varying slope for the effect of Prime and Priming Type per participant. We ran 4 MCMC chains, each with 11,000 iterations (including 1,000 warm up iterations), yielding a total of 40,000 posterior samples for statistical inference. For the effects of interest (as outlined in the research objectives and hypotheses), the mean of the posterior distribution and the Bayes factor (BF_10_) were reported, with the latter providing a measure of relative evidence for the alternative relative to the null hypotheses [(e.g., Wagenmakers, 2007)](https://www.zotero.org/google-docs/?BFw3iZ). Priors and model were specified as follows:

**Priors**

*priors <- c(*

*prior(normal(0.1, 0.5), class = "Intercept"),*

*prior(normal(0, 0.2), class = "b"),*

*prior(exponential(5), class = "sd"),*

*prior(normal(log(1.30), 0.3), class = "Intercept", dpar = "bs"),*

*prior(normal(0, 0.1), class = "b", dpar = "bs"),*

*prior(exponential(10), class = "sd", dpar = "bs"),*

*prior(normal(log(0.30), 0.10), class = "Intercept", dpar = "ndt"),*

*prior(normal(0, 0.05), class = "b", dpar = "ndt"),*

*prior(exponential(30), class = "sd", dpar = "ndt"),*

*prior(normal(0, 0.5), class = "Intercept", dpar = "bias"),*

*prior(normal(0, 0.2), class = "b", dpar = "bias"),*

*prior(exponential(5), class = "sd", dpar = "bias")*

*)*

**Model**

*RT | dec(Response) ~ 1 + Prime × Priming Type × Group × PC1 + (1 + Prime × Priming Type | Participant) + (1 | Target),*

*bs ~ 1 + Prime × Priming Type × Group × PC1 + (1 + Prime × Priming Type | Participant) + (1 | Target),*

*ndt ~ 1 + Prime × Priming Type × Group × PC1 + (1 + Prime × Priming Type | Participant) + (1 | Target),*

*bias ~ 1 + Prime × Priming Type × Group × PC1 + (1 + Prime × Priming Type | Participant) + (1 | Target)*

As for the mixed-effects models, the pre-specified DDM specification was compared with a version including Age and Sex as additional fixed effects using LOO-CV. The specification including Age and Sex predicted better and was retained.

**Contrasts**

All pairwise contrasts were estimated with the *emmeans* package, following the order of the factor levels (Prime: Control, Happy, Sad; Group: HC, BD, DEP; Priming Type: Masked, Unmasked). Contrasts are therefore reported as Control − Happy and Control − Sad for primes, HC − BD, HC − DEP and BD − DEP for groups, Masked − Unmasked for priming type.

## Supplementary Methods S8. Prime visibility task

All trials with prime durations < 25 ms or > 40 ms were excluded from the analyses of the prime visibility task.

(1) Visibility detection sensitivity (*d'*) was computed for each participant, following the standard formula: d’ = z(H) - z(FA), where H (Hit rate) is the proportion of hits among all prime-present trials, and FA (False Alarm rate) is the proportion of false alarms among all prime-absent trials. Higher d' values indicate better discrimination between signal (masked prime) and noise (no prime).

(2) Moreover, Happy/Sad d’ was computed for each participant using the same formula: d’ = z(H) - z(FA). In this case, H (Hit rate) represents the proportion of correctly identified happy or sad primes (i.e., positive and negative responses, respectively), while FA (False Alarm rate) represents the proportion of incorrect responses (misidentifications). A higher Happy/Sad d’ value reflects a greater ability to discriminate between happy and sad primes.

In both analyses, we applied log-linear correction to hit and false-alarm counts to keep d′ finite at extreme rates (Hautus, 1995).

We quantified evidence that *d′* exceeded chance within each group with Bayesian one-sample t-tests, and pairwise between-group differences with Bayesian independent-samples t-tests (*BayesFactor* package; Morey & Rouder, 2024). Posterior means, 95% credible intervals (from 10,000 posterior samples) and BF_10_ are reported.

To test the relationship between priming effects with prime discriminability, we computed each participant's RT priming effect on masked trials (incongruent − congruent), separately for unambiguous targets (correct trials only; congruence defined by prime–target valence match) and for ambiguous morph targets (congruence defined relative to the participant's own categorical response). Each was correlated with Happy/Sad d′ within each group using Bayesian correlation analyses; posterior median ρ, 95% credible intervals and BF_10_ are reported. This analysis addresses only whether the magnitude of priming scaled with individual differences in measured discriminability; it does not establish that priming effects were independent of prime awareness.

## Supplementary Methods S9. Correlation between Reactivity Index (PC3) and Reaction Time Priming Effects

PC3 reflected reactivity, distractibility, and hypersensitivity (Table S3, Figure S2), and was therefore interpreted as a reactivity index. Based on this, we hypothesized that higher PC3 scores would be associated with stronger RT priming effects. For each participant, we computed RT priming scores by subtracting RT for congruent from incongruent trials, separately for ambiguous and unambiguous targets, and for masked and unmasked primes. These four priming scores were then correlated with PC3 scores.

For patients, we applied the same analysis pipeline separately for BD and DEP.

# Supplementary Tables

## Supplementary Table S1. Demographic characteristics for HC, BD, and DEP.

| **Variable** | **HC (n=53)** | **BD (n=14)** | **DEP (n=17)** | **p-value** |
| --- | --- | --- | --- | --- |
| **Age, mean ± SD** | 32 ± 9.80 | 31 ± 9.33 | 31 ± 8.09 | p=.996 |
| **Female, n (%)** | 29 (55%) | 8 (57%) | 9 (53%) | p=.854 |

*Table S1.* Demographic characteristics for HC, BD and DEP. Age is presented as mean ± standard deviation (SD); sex is reported as number and percentage of female participants. Differences in age across groups were assessed using a one-way ANOVA (age ~ group), and sex distribution was compared using a chi-squared test. No significant group differences were found for age (*F*(2, 81) = 0.0037, p = .996) or sex distribution (χ²(2) = 0.32, p = .854).

## Supplementary Table S2. Summary of hypotheses and results.

| **Hypotheses** | **Main Results** | **Support** |
| --- | --- | --- |
| **(1) Priming effects on reaction times (RTs) to unambiguous emotional targets (happy/ sad)** |  | |
| *(1a) Faster RTs following congruent vs. incongruent primes* | Congruence effect in masked trials in all groups; and in unmasked trials in HC and BD, with evidence for no effect in DEP | Partially supported |
| *(1b) Stronger effects in BD than HC and DEP* | Evidence for no differences between groups, in both masked and unmasked trials | Not supported |
| *(1c) Stronger effects in unmasked > masked trials* | No differences between priming types in BD; inconclusive in HC and DEP | Not supported |
| **(2) Effects of mood and primes on the categorization of ambiguous targets** |  | |
| *(2a) Mood-congruent categorization bias* | No conclusive evidence for an effect of mood, across or within groups | Not supported |
| *(2b) Prime-induced categorization bias* | Happy primes shifted categorization toward positive in HC and BD, in both masked and unmasked trials; in DEP, only masked sad primes shifted categorization toward negative | Partially supported |
| *(2c) Stronger effects in BD than HC* | No conclusive evidence for a difference between HC and BD | Not supported |
| *(2d) Negativity bias in DEP (enhanced sad, reduced happy)* | Stronger effect of masked sad primes I DEP than HC and BD; evidence for no effect of happy primes in DEP (masked) | Partially supported |
| *(2e) Stronger effects in unmasked vs. masked trials* | No conclusive evidence for a difference, except a stronger effect of sad primes in masked vs. unmasked trials | Not supported |
| **(3) Priming effects on RTs to ambiguous targets** |  | |
| *(3a) Faster RTs following response-congruent prime* | Masked trials: effect in HC and BD, evidence for no effect in DEP. Unmasked trials: effects in BD only, evidence for no effect in HC and DEP | Partially supported |
| *(3b) Stronger effects in BD than HC and DEP* | Masked trials: stronger in BD than both HC and DEP. Unmasked: stronger in BD vs. HC, inconclusive vs. DEP | Partially supported |
| *(3c) Stronger effects in unmasked than masked trials* | Stronger in masked than unmasked in HC; no differences in BD and DEP | Not supported |
| **(4) Effects of mood and primes on decision making (DDM)** |  | |
| *(4a) Mood shifts in starting point and drift rate in a valence-congruent manner* | Evidence for no effect of mood on starting point; slight effect on drift rate | Not supported (starting point); Partially supported (drift rate) |
| *(4b) Primes shift starting point and drift rate in a valence-congruent manner* | Starting point: evidence for congruent shifts after masked happy and sad primes in HC and BD. Drift rate: effects present but inconsistent in direction across groups and priming types | Partially supported (starting point); Inconsistent (drift rate) |
| *(4c) Stronger prime-induced shifts in BD than HC* | No conclusive evidence for group differences | Not supported |
| *(4d) Negativity bias in DEP* | Starting point: no conclusive evidence. Drift rate: strong group differences driven by an unexpected direction in HC/BD; DEP showed a non-conclusive trend in the expected direction | Not supported |
| *(4e) Stronger effects in unmasked vs. masked trials* | Starting point: larger in masked than unmasked in HC (sad primes); no differences elsewhere. Drift rate: larger decrease in unmasked than masked after happy primes in all groups | Partially supported |

Each row corresponds to a prediction stated in the Introduction. "Supported" indicates conclusive evidence consistent with the prediction; "Partially supported" indicates that the prediction was confirmed for some conditions or groups but not others; "Not supported" indicates conclusive evidence against the prediction, in favour of the null, in the opposite direction to that predicted, that the evidence was insufficient to distinguish between H₁ and H₀.

## Supplementary Table S3. Questionnaire scores for HC, BD, and DEP.

| **Variables** | **HC (n=53)** | **BD (n=14)** | **DEP (n=17)** | **p-value** | **P-value post-hoc (Tukey corrected)** | | |
| --- | --- | --- | --- | --- | --- | --- | --- |
|  |  |  |  |  | **HC>BD** | **HC>DEP** | **BD>DEP** |
| **PANAS** | | | | | | | |
| *Positive Affect* | 27.11 ± 6.79 | 27.93 ± 8.97 | 27.00 ± 9.24 | p=.931 |  | | |
| *Negative Affect* | 18.26 ± 6.17 | 26.86 ± 8.55 | 24.71 ± 9.62 | **p<.001***** | **p=.001***** | **p=.007**** | p=.699 |
| **MAThyS - Part 1** | | | | | | | |
| *Thought Processes* | 22.62 ± 5.45 | 18.36 ± 6.19 | 17.85 ± 5.61 | **p<.003**** | **p<.035*** | **p<.009**** | p=.966 |
| *Emotional Reactivity* | 22.31 ± 5.62 | 22.61 ± 7.07 | 22.71 ± 8.45 | p=.971 |  | | |
| *Motivation* | 14.16 ± 4.12 | 17.11 ± 6.57 | 13.59 ± 5.77 | p=.100 |  | | |
| *Sensory perception* | 28.26 ± 4.78 | 27.43 ± 7.33 | 29.18 ± 6.49 | p=.688 |  | | |
| *Motor functions* | 18.13 ± 5.48 | 19.54 ± 8.64 | 13.74 ± 7.62 | **p=.028*** | p=.756 | **p=.047*** | **p=.042*** |
| **MAThyS - Part 2** | | | | | | | |
| *Sadness* | 2.25 ± 0.96 | 2.86 ± 0.95 | 3.12 ± 1.22 | **p=.005**** | p=.117 | **p=.008**** | p=.757 |
| *Joy* | 2.98 ± 1.07 | 2.71 ± 0.91 | 2.59 ± 0.94 | p=.330 |  | | |
| *Irritability* | 2.55 ± 0.97 | 2.93 ± 1.27 | 3.41 ± 1.28 | **p=.018*** | p=.478 | **p=.015*** | p=.440 |
| *Panic* | 1.68 ± 0.89 | 2.64 ± 1.28 | 2.53 ± 1.42 | **p=.002**** | **p=.011*** | p=.017 | p=.955 |
| *Anxiety* | 2.32 ± 0.96 | 3.43 ± 1.40 | 3.47 ± 1.42 | **p<.001***** | **p=.005**** | **p=.001**** | p=.994 |
| *Anger* | 1.87 ± 0.73 | 2.64 ± 1.22 | 2.41 ± 1.00 | **p=.006**** | **p=.013*** | p=.076. | p=750 |
| *Exaltation* | 2.09 ± 1.02 | 2.86 ± 1.23 | 2.12 ± 0.99 | p=.055. |  | | |

Questionnaire scores for HC, BD and DEP. Variables are presented as mean ± standard deviation (SD). Differences in age across groups were assessed using a one-way ANOVA (variable ~ group). Post-hoc tests were adjusted for multiple comparisons using the Tukey method.

## Supplementary Table S4. Feature contribution in PC1

| **Feature** | **Score** | **Scale** |
| --- | --- | --- |
| I am more interested in doing things | 0,2169 | MAThyS - Part 1 |
| I feel very energetic | 0,2144 | MAThyS - Part 1 |
| I am coming up with lots of new plans | 0,2135 | MAThyS - Part 1 |
| Enthusiastic | 0,2068 | PANAS |
| Joy | 0,2037 | MAThyS - Part 2 |
| I feel like communicating with other people more than usual | 0,2008 | MAThyS - Part 1 |
| Proud | 0,1878 | PANAS |
| Interested | 0,1836 | PANAS |
| Strong | 0,1833 | PANAS |
| Inspired | 0,1825 | PANAS |
| Determined | 0,1812 | PANAS |
| I feel outgoing | 0,1770 | MAThyS - Part 1 |
| Active | 0,1604 | PANAS |
| Alert | 0,1547 | PANAS |
| Excited | 0,1544 | PANAS |
| I like to eat because I appreciate how food tastes more than usual | 0,1459 | MAThyS - Part 1 |
| I feel like ideas are racing through my head | 0,1298 | MAThyS - Part 1 |
| Euphoria | 0,1214 | MAThyS - Part 2 |
| I am easily distracted and the slightest thing attracts my attention | 0,0678 | MAThyS - Part 1 |
| I am making decisions faster than usual | 0,0620 | MAThyS - Part 1 |
| I seem to be more sensitive to smells than usual | 0,0570 | MAThyS - Part 1 |
| I am more responsive than usual to things going on around me | 0,0531 | MAThyS - Part 1 |
| I feel restless or physically agitated | 0,0382 | MAThyS - Part 1 |
| I am more sensitive to touch than usual | 0,0365 | MAThyS - Part 1 |
| Alert | 0,0273 | PANAS |
| I am more sensitive to colours than usual | 0,0098 | MAThyS - Part 1 |
| I am particularly sensitive to music | 0,0079 | MAThyS - Part 1 |
| My emotions are very intense | -0,0075 | MAThyS - Part 1 |
| My mind never stops | -0,0099 | MAThyS - Part 1 |
| I have a lot of tension | -0,0198 | MAThyS - Part 1 |
| My mood seems to vary a lot, depending on my environment | -0,0242 | MAThyS - Part 1 |
| I lose control over my emotions sometimes | -0,0263 | MAThyS - Part 1 |
| Afraid | -0,0599 | PANAS |
| Upset | -0,0946 | PANAS |
| Guilty | -0,0966 | PANAS |
| Scared | -0,0969 | PANAS |
| Ashamed | -0,1138 | PANAS |
| Hostile | -0,1450 | PANAS |
| Distressed | -0,1489 | PANAS |
| Anger | -0,1644 | MAThyS - Part 2 |
| Jittery | -0,1744 | PANAS |
| Irritable | -0,1915 | PANAS |
| Panic | -0,1923 | MAThyS - Part 2 |
| Anxiety | -0,1965 | MAThyS - Part 2 |
| Irritability | -0,2007 | MAThyS - Part 2 |
| Sadness | -0,2062 | MAThyS - Part 2 |
| Nervous | -0,2247 | PANAS |

## Supplementary Table S5. Feature contribution in PC2

| **Feature** | **Score** | **Scale** |
| --- | --- | --- |
| Upset | 0,2415 | PANAS |
| Afraid | 0,2401 | PANAS |
| Scared | 0,2355 | PANAS |
| Ashamed | 0,2320 | PANAS |
| Excited | 0,2170 | PANAS |
| Euphoria | 0,2050 | MAThyS - Part 2 |
| Alert | 0,2048 | PANAS |
| Inspired | 0,1994 | PANAS |
| Distressed | 0,1987 | PANAS |
| Enthusiastic | 0,1889 | PANAS |
| Guilty | 0,1853 | PANAS |
| Irritable | 0,1847 | PANAS |
| Jittery | 0,1815 | PANAS |
| Panic | 0,1788 | MAThyS - Part 2 |
| Hostile | 0,1761 | PANAS |
| Anger | 0,1750 | MAThyS - Part 2 |
| Proud | 0,1670 | PANAS |
| Determined | 0,1654 | PANAS |
| Nervous | 0,1646 | PANAS |
| Irritability | 0,1644 | MAThyS - Part 2 |
| Strong | 0,1633 | PANAS |
| Active | 0,1524 | PANAS |
| Alert | 0,1348 | PANAS |
| I feel restless or physically agitated | 0,1334 | MAThyS - Part 1 |
| Anxiety | 0,1221 | MAThyS - Part 2 |
| I am coming up with lots of new plans | 0,1147 | MAThyS - Part 1 |
| I feel outgoing | 0,1111 | MAThyS - Part 1 |
| Sadness | 0,1063 | MAThyS - Part 2 |
| I am more sensitive to colours than usual | 0,1054 | MAThyS - Part 1 |
| I feel very energetic | 0,1054 | MAThyS - Part 1 |
| Interested | 0,0988 | PANAS |
| I lose control over my emotions sometimes | 0,0932 | MAThyS - Part 1 |
| I feel like ideas are racing through my head | 0,0884 | MAThyS - Part 1 |
| Joy | 0,0859 | MAThyS - Part 2 |
| I have a lot of tension | 0,0830 | MAThyS - Part 1 |
| I am more interested in doing things | 0,0579 | MAThyS - Part 1 |
| I seem to be more sensitive to smells than usual | 0,0415 | MAThyS - Part 1 |
| I am more responsive than usual to things going on around me | 0,0345 | MAThyS - Part 1 |
| I like to eat because I appreciate how food tastes more than usual | 0,0216 | MAThyS - Part 1 |
| My mood seems to vary a lot, depending on my environment | 0,0202 | MAThyS - Part 1 |
| I feel like communicating with other people more than usual | 0,0151 | MAThyS - Part 1 |
| My emotions are very intense | 0,0086 | MAThyS - Part 1 |
| My mind never stops | -0,0367 | MAThyS - Part 1 |
| I am making decisions faster than usual | -0,0468 | MAThyS - Part 1 |
| I am more sensitive to touch than usual | -0,0477 | MAThyS - Part 1 |
| I am easily distracted and the slightest thing attracts my attention | -0,0551 | MAThyS - Part 1 |
| I am particularly sensitive to music | -0,0879 | MAThyS - Part 1 |

## Supplementary Table S6. Feature contribution in PC3

| **Feature** | **Score** | **Scale** |
| --- | --- | --- |
| I am more responsive than usual to things going on around me | 0,4146 | MAThyS - Part 1 |
| My mind never stops | 0,3526 | MAThyS - Part 1 |
| I am particularly sensitive to music | 0,3389 | MAThyS - Part 1 |
| My emotions are very intense | 0,3229 | MAThyS - Part 1 |
| I am more sensitive to touch than usual | 0,2973 | MAThyS - Part 1 |
| My mood seems to vary a lot, depending on my environment | 0,2466 | MAThyS - Part 1 |
| I am easily distracted and the slightest thing attracts my attention | 0,2162 | MAThyS - Part 1 |
| I am making decisions faster than usual | 0,1391 | MAThyS - Part 1 |
| Interested | 0,1322 | PANAS |
| I lose control over my emotions sometimes | 0,1165 | MAThyS - Part 1 |
| Active | 0,1133 | PANAS |
| Alert | 0,1075 | PANAS |
| Anxiety | 0,0975 | MAThyS - Part 2 |
| Excited | 0,0888 | PANAS |
| Determined | 0,0826 | PANAS |
| Sadness | 0,0738 | MAThyS - Part 2 |
| Irritable | 0,0731 | PANAS |
| Jittery | 0,0665 | PANAS |
| Proud | 0,0663 | PANAS |
| Panic | 0,0623 | MAThyS - Part 2 |
| I have a lot of tension | 0,0607 | MAThyS - Part 1 |
| Enthusiastic | 0,0606 | PANAS |
| Nervous | 0,0544 | PANAS |
| I like to eat because I appreciate how food tastes more than usual | 0,0487 | MAThyS - Part 1 |
| Inspired | 0,0483 | PANAS |
| Irritability | 0,0462 | PANAS |
| Upset | 0,0422 | PANAS |
| Strong | 0,0313 | PANAS |
| Euphoria | 0,0282 | MAThyS - Part 2 |
| Alert | 0,0196 | PANAS |
| I feel like ideas are racing through my head | 0,0040 | MAThyS - Part 1 |
| Anger | -0,0186 | MAThyS - Part 2 |
| Ashamed | -0,0315 | PANAS |
| Joy | -0,0315 | MAThyS - Part 2 |
| I feel restless or physically agitated | -0,0383 | MAThyS - Part 1 |
| Guilty | -0,0425 | PANAS |
| Hostile | -0,0475 | PANAS |
| Scared | -0,0501 | PANAS |
| I am coming up with lots of new plans | -0,0863 | MAThyS - Part 1 |
| I feel like communicating with other people more than usual | -0,0865 | MAThyS - Part 1 |
| Distressed | -0,0927 | PANAS |
| I am more interested in doing things | -0,1020 | MAThyS - Part 1 |
| I seem to be more sensitive to smells than usual | -0,1140 | MAThyS - Part 1 |
| I am more sensitive to colours than usual | -0,1217 | MAThyS - Part 1 |
| I feel very energetic | -0,1244 | MAThyS - Part 1 |
| Afraid | -0,1678 | PANAS |
| I feel outgoing | -0,1715 | MAThyS - Part 1 |

## Supplementary Table S7. Feature contribution in PC4

| **Feature** | **Score** | **Scale** |
| --- | --- | --- |
| I have a lot of tension | 0,4117 | MAThyS - Part 1 |
| I lose control over my emotions sometimes | 0,3187 | MAThyS - Part 1 |
| I like to eat because I appreciate how food tastes more than usual | 0,2310 | MAThyS - Part 1 |
| My emotions are very intense | 0,2192 | MAThyS - Part 1 |
| I feel like ideas are racing through my head | 0,1931 | MAThyS - Part 1 |
| I seem to be more sensitive to smells than usual | 0,1913 | MAThyS - Part 1 |
| I am making decisions faster than usual | 0,1854 | MAThyS - Part 1 |
| Joy | 0,1822 | PANAS |
| I am particularly sensitive to music | 0,1594 | MAThyS - Part 1 |
| I feel outgoing | 0,1582 | MAThyS - Part 1 |
| I feel very energetic | 0,1537 | MAThyS - Part 1 |
| Afraid | 0,1499 | PANAS |
| Distressed | 0,1496 | PANAS |
| I am coming up with lots of new plans | 0,1327 | MAThyS - Part 1 |
| I am more sensitive to colours than usual | 0,1256 | MAThyS - Part 1 |
| My mind never stops | 0,1219 | MAThyS - Part 1 |
| I feel restless or physically agitated | 0,1004 | MAThyS - Part 1 |
| I feel like communicating with other people more than usual | 0,0952 | MAThyS - Part 1 |
| I am easily distracted and the slightest thing attracts my attention | 0,0889 | MAThyS - Part 1 |
| Guilty | 0,0703 | PANAS |
| Scared | 0,0652 | PANAS |
| Euphoria | 0,0597 | MAThyS - Part 2 |
| Irritable | 0,0579 | PANAS |
| Nervous | 0,0524 | PANAS |
| I am more interested in doing things | 0,0469 | MAThyS - Part 1 |
| Upset | 0,0096 | PANAS |
| I am more responsive than usual to things going on around me | 0,0068 | MAThyS - Part 1 |
| Anger | 0,0000 | MAThyS - Part 2 |
| Ashamed | -0,0009 | PANAS |
| Jittery | -0,0029 | PANAS |
| My mood seems to vary a lot, depending on my environment | -0,0051 | MAThyS - Part 1 |
| Panic | -0,0076 | MAThyS - Part 2 |
| I am more sensitive to touch than usual | -0,0140 | MAThyS - Part 1 |
| Excited | -0,0332 | PANAS |
| Hostile | -0,0413 | PANAS |
| Sadness | -0,0568 | MAThyS - Part 2 |
| Anxiety | -0,1047 | MAThyS - Part 2 |
| Enthusiastic | -0,1114 | PANAS |
| Interested | -0,1190 | PANAS |
| Strong | -0,1243 | PANAS |
| Irritability | -0,1337 | MAThyS - Part 2 |
| Alert | -0,1375 | PANAS |
| Inspired | -0,1456 | PANAS |
| Active | -0,1783 | PANAS |
| Proud | -0,1787 | PANAS |
| Determined | -0,2020 | PANAS |
| Alert | -0,2210 | PANAS |

## Supplementary Table S8. Proportion of correct responses per Priming Type, Group, Congruence, and Target

| **Priming Type** | **Group** | **Congruence** | **Target** | **Number of trials** | **Proportion correct** |
| --- | --- | --- | --- | --- | --- |
| **Masked** | **BD** | **Congruent** | **Happy** | 460 | 0.958696 |
|  |  |  | **Sad** | 469 | 0.963753 |
|  |  | **Incongruent** | **Happy** | 468 | 0.952991 |
|  |  |  | **Sad** | 466 | 0.969957 |
|  | **HC** | **Congruent** | **Happy** | 1821 | 0.956068 |
|  |  |  | **Sad** | 1821 | 0.964854 |
|  |  | **Incongruent** | **Happy** | 1816 | 0.946035 |
|  |  |  | **Sad** | 1797 | 0.94936 |
|  | **DEP** | **Congruent** | **Happy** | 574 | 0.944251 |
|  |  |  | **Sad** | 584 | 0.955479 |
|  |  | **Incongruent** | **Happy** | 585 | 0.916239 |
|  |  |  | **Sad** | 576 | 0.961806 |
| **Unmasked** | **BD** | **Congruent** | **Happy** | 470 | 0.965957 |
|  |  |  | **Sad** | 468 | 0.976496 |
|  |  | **Incongruent** | **Happy** | 465 | 0.954839 |
|  |  |  | **Sad** | 470 | 0.96383 |
|  | **HC** | **Congruent** | **Happy** | 1817 | 0.952119 |
|  |  |  | **Sad** | 1837 | 0.964616 |
|  |  | **Incongruent** | **Happy** | 1827 | 0.953476 |
|  |  |  | **Sad** | 1813 | 0.957529 |
|  | **DEP** | **Congruent** | **Happy** | 580 | 0.937931 |
|  |  |  | **Sad** | 577 | 0.961872 |
|  |  | **Incongruent** | **Happy** | 574 | 0.933798 |
|  |  |  | **Sad** | 583 | 0.962264 |

Note. "Number of trials" is the total number of trials in each cell, summed across all participants in the group after trial-level exclusions. Because group sizes differ (HC n = 53, BD n = 14, DEP n = 17), these totals differ accordingly. "Proportion correct" is the mean proportion of correct responses in each cell.

## Supplementary Table S9. Model comparison for reaction times to unambiguous targets

| **Model** | **ELPD LOO** | **Δ ELPD** | **LOOIC** |
| --- | --- | --- | --- |
| **1** | 16442.651 (174.79) | 0 (0) | -32885.303 (349.58) |
| **2** | -16442.396 (174.84) | -0.255 (5.097) | 1-32884.793 (349.68) |

Model 1 included Congruence (Congruent, Incongruent), Group (HC, BD, DEP), Priming Type (Masked, Unmasked), and their interaction as fixed factors, and Target (Happy, Sad) as an additional covariate, with random effects included by-participant intercepts and slopes for Congruence × Priming Type, and by-target random intercepts and slopes. Model 2 added Age and Sex as an additional fixed factor. Higher ELPD values and lower LOOIC values indicate better out-of-sample predictive performance.

## Supplementary Table S10. Bayesian estimates of priming effects on reaction times (Congruent – Incongruent) for unambiguous targets across groups and priming type

| **Group** | **Priming Type** | **Prime Contrast** | **Estimate** | **Change RT (%)** | **CrI** | **BF_10_** | **ROPE (%)** |
| --- | --- | --- | --- | --- | --- | --- | --- |
| **BD** | **Masked** | **Congruent - Incongruent** | -0.049 | -4.76% | (-0.07, -0.03) | **182.48** | 0.26% |
|  | **Unmasked** | **Congruent - Incongruent** | -0.045 | -4.38% | (-0.07, -0.02) | **97.46** | 0.58% |
| **HC** | **Masked** | **Congruent - Incongruent** | -0.049 | -4.76% | (-0.06, -0.04) | **9.37 × 10⁶** | 0% |
|  | **Unmasked** | **Congruent - Incongruent** | -0.031 | -3.08% | (-0.04, -0.02) | **2.59 × 10³** | 0.5% |
| **DEP** | **Masked** | **Congruent - Incongruent** | -0.041 | -4.06% | (-0.06, -0.02) | **87** | 0.83% |
|  | **Unmasked** | **Congruent - Incongruent** | -0.019 | -1.84% | (-0.04, 0.00) | **0.33** | 40.31% |

*Estimate: posterior mean of the congruence effect on log-RT; Change RT (%): percentage change in RT on the original scale; CrI: 95% credibility interval for the posterior mean; BF₁₀: Bayes Factor in favor of the alternative hypothesis (values > 3 are shown in bold, indicating at least moderate evidence; values < 0.3 are shown in bold, indicating at least moderate evidence in favor of the null hypothesis); ROPE (%): percentage of the posterior distribution falling within the region of practical equivalence.*

## Supplementary Table S11. Bayesian estimates of priming effects on reaction times (Congruent – Incongruent) for unambiguous targets between groups and across priming type

| **Group Contrast** | **Priming Type** | **Prime Contrast** | **Estimate** | **Change RT (%)** | **CrI** | **BF_10_** | **ROPE (%)** |
| --- | --- | --- | --- | --- | --- | --- | --- |
| **BD - DEP** | **Masked** | **Congruent - Incongruent** | -0.007 | -0.74% | (-0.04, 0.02) | **0.11** | 64.26% |
|  | **Unmasked** | **Congruent - Incongruent** | -0.026 | -2.59% | (-0.06, 0.00) | 0.4 | 25% |
| **HC - BD** | **Masked** | **Congruent - Incongruent** | 0 | 0% | (-0.03, 0.03) | **0.12** | 78.33% |
|  | **Unmasked** | **Congruent - Incongruent** | 0.014 | 1.37% | (-0.01, 0.04) | **0.2** | 56.41% |
| **HC - DEP** | **Masked** | **Congruent - Incongruent** | -0.007 | -0.74% | (-0.03, 0.02) | **0.13** | 73.69% |
|  | **Unmasked** | **Congruent - Incongruent** | -0.013 | -1.26% | (-0.04, 0.01) | **0.19** | 60.25% |

*Estimate: posterior mean of the congruence effect on log-RT; Change RT (%): percentage change in RT on the original scale; CrI: 95% credibility interval for the posterior mean; BF₁₀: Bayes Factor in favor of the alternative hypothesis (values > 3 are shown in bold, indicating at least moderate evidence; values < 0.3 are shown in bold, indicating at least moderate evidence in favor of the null hypothesis); ROPE (%): percentage of the posterior distribution falling within the region of practical equivalence.*

## Supplementary Table S12. Bayesian estimates of priming effects on reaction times (Congruent – Incongruent) for unambiguous targets between priming type and across group

| **Group** | **Priming Type Contrast** | **Estimate** | **Change RT (%)** | **CrI** | **BF_10_** | **ROPE (%)** |
| --- | --- | --- | --- | --- | --- | --- |
| **BD** | **Unmasked - Masked** | 0.004 | 0.4% | (-0.02, 0.03) | **0.09** | 77.12% |
| **HC** | **Unmasked - Masked** | 0.018 | 1.77% | (0.00, 0.03) | **2.49** | 40.57% |
| **DEP** | **Unmasked - Masked** | 0.023 | 2.31% | (0.00, 0.05) | 0.58 | 27.79% |

*Estimate: posterior mean of the congruence effect on log-RT; Change RT (%): percentage change in RT on the original scale; CrI: 95% credibility interval for the posterior mean; BF₁₀: Bayes Factor in favor of the alternative hypothesis (values > 3 are shown in bold, indicating at least moderate evidence; values < 0.3 are shown in bold, indicating at least moderate evidence in favor of the null hypothesis); ROPE (%): percentage of the posterior distribution falling within the region of practical equivalence.*

## Supplementary Table S13. Bayesian estimates of priming effects on reaction times (Congruent – Incongruent) for unambiguous targets between target valence across groups and priming type

| **Priming Type** | **Group** | **Target Valence Contrast** | **Prime Contrast** | **Estimate** | **Change RT (%)** | **CrI** | **BF_10_** | **ROPE (%)** |
| --- | --- | --- | --- | --- | --- | --- | --- | --- |
| **Masked** | **BD** | **Happy - Sad** | **Congruent - Incongruent** | -0.028 | -2.72% | (-0.06, 0.01) | 0.39 | 24.72% |
|  | **HC** | **Happy - Sad** | **Congruent - Incongruent** | -0.015 | -1.53% | (-0.03, 0.00) | 0.36 | 52.59% |
|  | **DEP** | **Happy - Sad** | **Congruent - Incongruent** | -0.003 | -0.26% | (-0.03, 0.03) | **0.1** | 69.01% |
| **Unmasked** | **BD** | **Happy - Sad** | **Congruent - Incongruent** | -0.021 | -2.08% | (-0.05, 0.01) | **0.23** | 37.35% |
|  | **HC** | **Happy - Sad** | **Congruent - Incongruent** | -0.011 | -1.13% | (-0.03, 0.01) | **0.18** | 69.78% |
|  | **DEP** | **Happy - Sad** | **Congruent - Incongruent** | -0.001 | -0.15% | (-0.03, 0.03) | **0.1** | 68.29% |

*Estimate: posterior mean of the congruence effect on log-RT; Change RT (%): percentage change in RT on the original scale; CrI: 95% credibility interval for the posterior mean; BF₁₀: Bayes Factor in favor of the alternative hypothesis (values > 3 are shown in bold, indicating at least moderate evidence; values < 0.3 are shown in bold, indicating at least moderate evidence in favor of the null hypothesis); ROPE (%): percentage of the posterior distribution falling within the region of practical equivalence.*

## Supplementary Table S14. Model comparison for responses to ambiguous targets

| **Model** | **ELPD LOO** | **Δ ELPD** | **LOOIC** |
| --- | --- | --- | --- |
| **1** | -7411.379 (75.40) | 0.000 (0.000) | 14822.757 (150.80) |
| **2** | -7411.527 (75.40) | -0.148 (0.382) | 14823.053 (150.80) |

Model 1 included Prime (Happy, Sad, Control), Group (HC, BD, DEP), Priming Type (Masked, Unmasked), Mood, and their interactions as fixed factor, with random effects included by-participant intercepts and slopes for Prime × Priming Type, and by-target random intercepts and slopes. Model 2 added Age and Sex as an additional fixed factor. Higher ELPD values and lower LOOIC values indicate better out-of-sample predictive performance.

## Supplementary Table S15. Bayesian estimates for the effects of mood (as indexed by PC1) on morph responses

| **Prime** | **Estimate** | **CrI** | **BF_10_** | **ROPE (%)** |
| --- | --- | --- | --- | --- |
| **Control** | 0.139 | (0.00, 0.28) | **0.9** | 72.16% |

*Estimate: posterior mean of the prime effect on the parameter; CrI: 95% credibility interval for the posterior mean; BF₁₀: Bayes Factor in favor of the alternative hypothesis (values > 3 are shown in bold, indicating at least moderate evidence; values < 0.3 are shown in bold, indicating at least moderate evidence in favor of the null hypothesis); ROPE (%): percentage of the posterior distribution falling within the region of practical equivalence.*

## Supplementary Table S16. Bayesian estimates for the effects of mood (as indexed by PC1) on morph responses across groups

| **Prime** | **Group** | **Estimate** | **CrI** | **BF_10_** | **ROPE (%)** |
| --- | --- | --- | --- | --- | --- |
| **Control** | **BD** | 0.009 | (-0.27, 0.28) | **0.2** | 79.71% |
|  | **HC** | 0.197 | (-0.00, 0.40) | 1.32 | 42.88% |
|  | **DEP** | 0.211 | (-0.02, 0.43) | 0.93 | 39.52% |

*Estimate: posterior mean of the prime effect on the parameter; CrI: 95% credibility interval for the posterior mean; BF₁₀: Bayes Factor in favor of the alternative hypothesis (values > 3 are shown in bold, indicating at least moderate evidence; values < 0.3 are shown in bold, indicating at least moderate evidence in favor of the null hypothesis); ROPE (%): percentage of the posterior distribution falling within the region of practical equivalence.*

## Supplementary Table S17. Bayesian estimates for the effects of primes on morph responses across groups and priming type

| **Group** | **Priming Type** | **Prime Contrast** | **Estimate** | **CrI** | **BF_10_** | **ROPE (%)** |
| --- | --- | --- | --- | --- | --- | --- |
| **BD** | **Masked** | **Control - Happy** | -0.528 | (-0.90, -0.15) | **11.24** | 3.36% |
|  |  | **Control - Sad** | -0.148 | (-0.51, 0.19) | **0.32** | 53.83% |
|  | **Unmasked** | **Control - Happy** | -0.779 | (-1.15, -0.40) | **325.23** | 0.12% |
|  |  | **Control - Sad** | -0.131 | (-0.48, 0.22) | **0.3** | 57.18% |
| **HC** | **Masked** | **Control - Happy** | -0.228 | (-0.42, -0.03) | **2.22** | 32.02% |
|  |  | **Control - Sad** | -0.008 | (-0.20, 0.18) | **0.18** | 92.9% |
|  | **Unmasked** | **Control - Happy** | -0.481 | (-0.68, -0.28) | **2.33 × 10³** | 0.2% |
|  |  | **Control - Sad** | -0.207 | (-0.40, -0.02) | 1.63 | 39.07% |
| **DEP** | **Masked** | **Control - Happy** | 0.085 | (-0.26, 0.43) | **0.25** | 64.24% |
|  |  | **Control - Sad** | 0.412 | (0.09, 0.74) | **4.5** | 8.2% |
|  | **Unmasked** | **Control - Happy** | -0.354 | (-0.69, -0.02) | 1.82 | 15.32% |
|  |  | **Control - Sad** | -0.158 | (-0.49, 0.17) | **0.33** | 53.32% |

*Estimate: posterior mean of the prime effect on the parameter; CrI: 95% credibility interval for the posterior mean; BF₁₀: Bayes Factor in favor of the alternative hypothesis (values > 3 are shown in bold, indicating at least moderate evidence; values < 0.3 are shown in bold, indicating at least moderate evidence in favor of the null hypothesis); ROPE (%): percentage of the posterior distribution falling within the region of practical equivalence.*

## Supplementary Table S18. Bayesian estimates for the effects of primes on morph responses between groups across priming type

| **Prime Contrast** | **Group Contrast** | **Priming Type** | **Estimate** | **CrI** | **BF_10_** | **ROPE (%)** |
| --- | --- | --- | --- | --- | --- | --- |
| **Control - Happy** | **BD - DEP** | **Masked** | -0.613 | (-1.11, -0.11) | **5.94** | 4.33% |
|  |  | **Unmasked** | -0.426 | (-0.93, 0.08) | 1.3 | 16.01% |
|  | **HC - BD** | **Masked** | 0.3 | (-0.11, 0.70) | 1.09 | 26.67% |
|  |  | **Unmasked** | 0.298 | (-0.12, 0.70) | 1.04 | 27.09% |
|  | **HC - DEP** | **Masked** | -0.313 | (-0.70, 0.06) | 1.31 | 23.9% |
|  |  | **Unmasked** | -0.127 | (-0.51, 0.25) | 0.43 | 54.98% |
| **Control - Sad** | **BD - DEP** | **Masked** | -0.56 | (-1.02, -0.07) | **4.49** | 5.61% |
|  |  | **Unmasked** | 0.027 | (-0.44, 0.50) | **0.3** | 54% |
|  | **HC - BD** | **Masked** | 0.14 | (-0.24, 0.53) | 0.45 | 53% |
|  |  | **Unmasked** | -0.077 | (-0.46, 0.31) | 0.37 | 60.78% |
|  | **HC - DEP** | **Masked** | -0.42 | (-0.78, -0.06) | **4.11** | 9.81% |
|  |  | **Unmasked** | -0.05 | (-0.41, 0.32) | **0.33** | 65.38% |

*Estimate: posterior mean of the prime effect on the parameter; CrI: 95% credibility interval for the posterior mean; BF₁₀: Bayes Factor in favor of the alternative hypothesis (values > 3 are shown in bold, indicating at least moderate evidence; values < 0.3 are shown in bold, indicating at least moderate evidence in favor of the null hypothesis); ROPE (%): percentage of the posterior distribution falling within the region of practical equivalence.*

## Supplementary Table S19. Bayesian estimates for the effects of primes on morph responses between priming type across groups

| **Prime Contrast** | **Group** | **Priming Type Contrast** | **Estimate** | **CrI** | **BF_10_** | **ROPE (%)** |
| --- | --- | --- | --- | --- | --- | --- |
| **Control - Happy** | **BD** | **Masked - Unmasked** | 0.252 | (-0.21, 0.70) | 0.59 | 34.44% |
|  | **HC** | **Masked - Unmasked** | 0.253 | (0.00, 0.50) | 1.9 | 27.96% |
|  | **DEP** | **Masked - Unmasked** | 0.439 | (0.02, 0.85) | **2.56** | 10.87% |
| **Control - Sad** | **BD** | **Masked - Unmasked** | -0.017 | (-0.45, 0.42) | **0.31** | 57.83% |
|  | **HC** | **Masked - Unmasked** | 0.199 | (-0.04, 0.45) | 0.9 | 43.59% |
|  | **DEP** | **Masked - Unmasked** | 0.57 | (0.16, 0.99) | **11.53** | 3.23% |

*Estimate: posterior mean of the prime effect on the parameter; CrI: 95% credibility interval for the posterior mean; BF₁₀: Bayes Factor in favor of the alternative hypothesis (values > 3 are shown in bold, indicating at least moderate evidence; values < 0.3 are shown in bold, indicating at least moderate evidence in favor of the null hypothesis); ROPE (%): percentage of the posterior distribution falling within the region of practical equivalence.*

## Supplementary Table S20. Model comparison for reaction times to ambiguous targets

| **Model** | **ELPD LOO** | **Δ ELPD** | **LOOIC** |
| --- | --- | --- | --- |
| **2** | 4963.055 (142.93) | 0.000 (0.000) | -9926.111 (285.87) |
| **1** | 4962.650 (142.94) | -0.406 (0.410) | -9925.299 (285.88) |

Model 1 included Congruence (Congruent, Incongruent), Group (HC, BD, DEP), Priming Type (Masked, Unmasked), and their interactions as fixed factor, Response Valence (Positive, Negative) as an additional covariate, with random effects included by-participant intercepts and slopes for Congruence × Priming Type, and by-target random intercepts and slopes. Model 2 added Age and Sex as an additional fixed factor. Higher ELPD values and lower LOOIC values indicate better out-of-sample predictive performance.

## Supplementary Table S21. Bayesian estimates of priming effects on reaction times (Congruent – Incongruent) for ambiguous targets across groups and priming type

| **Group** | **Priming Type** | **Prime Contrast** | **Estimate** | **Change RT (%)** | **CrI** | **BF_10_** | **ROPE (%)** |
| --- | --- | --- | --- | --- | --- | --- | --- |
| **BD** | **Masked** | **Congruent - Incongruent** | -0.095 | -9.08% | (-0.13, -0.06) | **1.28 × 10⁴** | 0% |
|  | **Unmasked** | **Congruent - Incongruent** | -0.054 | -5.26% | (-0.08, -0.02) | **17.54** | 0.62% |
| **HC** | **Masked** | **Congruent - Incongruent** | -0.051 | -4.98% | (-0.07, -0.04) | **4.60 × 10⁵** | 0% |
|  | **Unmasked** | **Congruent - Incongruent** | -0.008 | -0.75% | (-0.02, 0.01) | **0.1** | 78.75% |
| **DEP** | **Masked** | **Congruent - Incongruent** | -0.006 | -0.64% | (-0.04, 0.02) | **0.09** | 62.19% |
|  | **Unmasked** | **Congruent - Incongruent** | -0.02 | -2% | (-0.05, 0.01) | **0.22** | 32.39% |

*Estimate: posterior mean of the congruence effect on log-RT; Change RT (%): percentage change in RT on the original scale; CrI: 95% credibility interval for the posterior mean; BF₁₀: Bayes Factor in favor of the alternative hypothesis (values > 3 are shown in bold, indicating at least moderate evidence; values < 0.3 are shown in bold, indicating at least moderate evidence in favor of the null hypothesis); ROPE (%): percentage of the posterior distribution falling within the region of practical equivalence.*

## Supplementary Table S22. Bayesian estimates of priming effects on reaction times (Congruent – Incongruent) for ambiguous targets between groups and across priming type

| **Group Contrast** | **Priming Type** | **Prime Contrast** | **Estimate** | **Change RT (%)** | **CrI** | **BF_10_** | **ROPE (%)** |
| --- | --- | --- | --- | --- | --- | --- | --- |
| **BD - DEP** | **Masked** | **Congruent - Incongruent** | -0.089 | -8.49% | (-0.13, -0.05) | **509.26** | 0.01% |
|  | **Unmasked** | **Congruent - Incongruent** | -0.034 | -3.32% | (-0.08, 0.01) | 0.42 | 16.27% |
| **HC - BD** | **Masked** | **Congruent - Incongruent** | 0.044 | 4.5% | (0.01, 0.08) | **3.22** | 4.51% |
|  | **Unmasked** | **Congruent - Incongruent** | 0.046 | 4.75% | (0.01, 0.08) | **4.68** | 3.23% |
| **HC - DEP** | **Masked** | **Congruent - Incongruent** | -0.045 | -4.37% | (-0.08, -0.01) | **5.75** | 3.2% |
|  | **Unmasked** | **Congruent - Incongruent** | 0.013 | 1.27% | (-0.02, 0.04) | **0.18** | 47.84% |

*Estimate: posterior mean of the congruence effect on log-RT; Change RT (%): percentage change in RT on the original scale; CrI: 95% credibility interval for the posterior mean; BF₁₀: Bayes Factor in favor of the alternative hypothesis (values > 3 are shown in bold, indicating at least moderate evidence; values < 0.3 are shown in bold, indicating at least moderate evidence in favor of the null hypothesis); ROPE (%): percentage of the posterior distribution falling within the region of practical equivalence.*

## Supplementary Table S23. Bayesian estimates of priming effects on reaction times (Congruent – Incongruent) for ambiguous targets between priming type and across group

| **Group** | **Priming Type Contrast** | **Estimate** | **Change RT (%)** | **CrI** | **BF_10_** | **ROPE (%)** |
| --- | --- | --- | --- | --- | --- | --- |
| **BD** | **Unmasked - Masked** | 0.041 | 4.2% | (-0.00, 0.08) | 0.82 | 9.86% |
| **HC** | **Unmasked - Masked** | 0.044 | 4.45% | (0.02, 0.07) | **108.76** | 0.32% |
| **DEP** | **Unmasked - Masked** | -0.014 | -1.37% | (-0.05, 0.02) | **0.16** | 41.98% |

*Estimate: posterior mean of the congruence effect on log-RT; Change RT (%): percentage change in RT on the original scale; CrI: 95% credibility interval for the posterior mean; BF₁₀: Bayes Factor in favor of the alternative hypothesis (values > 3 are shown in bold, indicating at least moderate evidence; values < 0.3 are shown in bold, indicating at least moderate evidence in favor of the null hypothesis); ROPE (%): percentage of the posterior distribution falling within the region of practical equivalence.*

## Supplementary Table S24. Bayesian estimates of priming effects on reaction times (Congruent – Incongruent) for ambiguous targets between target valence across groups and priming type

| **Priming Type** | **Group** | **Target Valence Contrast** | **Prime Contrast** | **Estimate** | **Change RT (%)** | **CrI** | **BF_10_** | **ROPE (%)** |
| --- | --- | --- | --- | --- | --- | --- | --- | --- |
| **Masked** | **BD** | **Negative - Positive** | **Congruent - Incongruent** | 0.007 | 0.67% | (-0.05, 0.06) | **0.19** | 36.04% |
|  | **HC** | **Negative - Positive** | **Congruent - Incongruent** | 0.012 | 1.23% | (-0.02, 0.04) | **0.19** | 50.98% |
|  | **DEP** | **Negative - Positive** | **Congruent - Incongruent** | 0 | -0.04% | (-0.05, 0.05) | **0.17** | 39.71% |
| **Unmasked** | **BD** | **Negative - Positive** | **Congruent - Incongruent** | 0.061 | 6.34% | (0.00, 0.12) | 1.64 | 4.72% |
|  | **HC** | **Negative - Positive** | **Congruent - Incongruent** | 0.038 | 3.86% | (0.01, 0.07) | **3.6** | 5.34% |
|  | **DEP** | **Negative - Positive** | **Congruent - Incongruent** | 0.053 | 5.45% | (0.00, 0.10) | 1.23 | 6.69% |

*Estimate: posterior mean of the congruence effect on log-RT; Change RT (%): percentage change in RT on the original scale; CrI: 95% credibility interval for the posterior mean; BF₁₀: Bayes Factor in favor of the alternative hypothesis (values > 3 are shown in bold, indicating at least moderate evidence; values < 0.3 are shown in bold, indicating at least moderate evidence in favor of the null hypothesis); ROPE (%): percentage of the posterior distribution falling within the region of practical equivalence.*

## Supplementary Table S25. Model comparison for drift diffusion models

| **Model** | **ELPD LOO** | **Δ ELPD** | **LOOIC** |
| --- | --- | --- | --- |
| **2** | -402.586 (133.74) | 0.000 (0.000) | 805.172 (262.49) |
| **1** | -407.982 (133.80) | -5.396 (2.653) | 815.964 (267.60) |

Model 1 included Prime (Happy, Sad, Control), Group (HC, BD, DEP), Priming Type (Masked, Unmasked), Mood (PC1), and their interactions as fixed factor, with random effects included included by-participant intercepts and slopes for Congruence × Priming Type, and by-target random intercepts and slopes. Model 2 added Age and Sex as an additional fixed factor. Higher ELPD values and lower LOOIC values indicate better out-of-sample predictive performance.

## Supplementary Table S26. Drift Diffusion Modeling results for the effect of primes on starting point and drift rate across groups and priming type

| **Group** | **Parameter** | **Priming Type** | **Prime Contrast** | **Estimate** | **CrI** | **BF_10_** | **ROPE (%)** |
| --- | --- | --- | --- | --- | --- | --- | --- |
| **HC** | **Starting Point** | **Masked** | **Control-Happy** | -0.218 | (-0.32, -0.11) | **304.66** | 1.49% |
|  |  |  | **Control-Sad** | 0.148 | (0.04, 0.25) | **10.46** | 18.56% |
|  |  | **Unmasked** | **Control-Happy** | -0.096 | (-0.20, 0.01) | 1.2 | 52.75% |
|  |  |  | **Control-Sad** | -0.05 | (-0.15, 0.05) | 0.37 | 82.32% |
|  | **Drift Rate** | **Masked** | **Control-Happy** | 0.036 | (-0.11, 0.19) | 0.37 | 76.86% |
|  |  |  | **Control-Sad** | -0.12 | (-0.27, 0.03) | 1.23 | 39.28% |
|  |  | **Unmasked** | **Control-Happy** | -0.25 | (-0.40, -0.10) | **58.81** | 2.6% |
|  |  |  | **Control-Sad** | -0.165 | (-0.31, -0.02) | **3.58** | 19.28% |
| **BD** | **Starting Point** | **Masked** | **Control-Happy** | -0.345 | (-0.54, -0.15) | **110.41** | 0.69% |
|  |  |  | **Control-Sad** | 0.279 | (0.10, 0.47) | **27.1** | 2.67% |
|  |  | **Unmasked** | **Control-Happy** | -0.193 | (-0.38, 0.00) | **2.09** | 17.11% |
|  |  |  | **Control-Sad** | 0.144 | (-0.04, 0.33) | 0.95 | 31.87% |
|  | **Drift Rate** | **Masked** | **Control-Happy** | 0.009 | (-0.25, 0.26) | 0.42 | 55.54% |
|  |  |  | **Control-Sad** | -0.277 | (-0.52, -0.02) | **4.31** | 8.26% |
|  |  | **Unmasked** | **Control-Happy** | -0.338 | (-0.60, -0.08) | **10.2** | 3.84% |
|  |  |  | **Control-Sad** | -0.219 | (-0.47, 0.03) | 1.81 | 16.74% |
| **DEP** | **Starting Point** | **Masked** | **Control-Happy** | -0.196 | (-0.38, -0.01) | **2.51** | 15.21% |
|  |  |  | **Control-Sad** | -0.014 | (-0.20, 0.16) | **0.29** | 72.03% |
|  |  | **Unmasked** | **Control-Happy** | -0.213 | (-0.39, -0.03) | **4.07** | 11.24% |
|  |  |  | **Control-Sad** | -0.098 | (-0.28, 0.08) | 0.52 | 49.09% |
|  | **Drift Rate** | **Masked** | **Control-Happy** | 0.177 | (-0.07, 0.43) | 1.04 | 25.84% |
|  |  |  | **Control-Sad** | 0.226 | (-0.02, 0.48) | 1.95 | 15.58% |
|  |  | **Unmasked** | **Control-Happy** | -0.122 | (-0.38, 0.13) | 0.61 | 39.3% |
|  |  |  | **Control-Sad** | -0.041 | (-0.28, 0.21) | 0.41 | 54.95% |

*Estimate: posterior mean of the prime effect on the parameter; CrI: 95% credibility interval for the posterior mean; BF₁₀: Bayes Factor in favor of the alternative hypothesis (values > 3 are shown in bold, indicating at least moderate evidence; values < 0.3 are shown in bold, indicating at least moderate evidence in favor of the null hypothesis); ROPE (%): percentage of the posterior distribution falling within the region of practical equivalence.*

## Supplementary Table S27. Drift Diffusion Modeling results for the effect of primes on starting point and drift rate between groups across priming type

| **Parameter** | **Priming Type** | **Prime Contrast** | **Group Contrast** | **Estimate** | **CrI** | **BF_10_** | **ROPE (%)** |
| --- | --- | --- | --- | --- | --- | --- | --- |
| **Starting Point** | **Masked** | **Control - Happy** | **HC - BD** | 0.127 | (-0.08, 0.33) | 1 | 37.97% |
|  |  | **Control - Happy** | **HC - DEP** | -0.022 | (-0.22, 0.17) | 0.45 | 67.66% |
|  |  | **Control - Happy** | **BD - DEP** | -0.149 | (-0.41, 0.11) | 0.79 | 32.38% |
|  |  | **Control - Sad** | **HC - BD** | -0.131 | (-0.32, 0.06) | 1.07 | 36.42% |
|  |  | **Control - Sad** | **HC - DEP** | 0.162 | (-0.02, 0.35) | 1.82 | 25.39% |
|  |  | **Control - Sad** | **BD - DEP** | 0.294 | (0.04, 0.53) | **6.1** | 6.25% |
|  | **Unmasked** | **Control - Happy** | **HC - BD** | 0.097 | (-0.11, 0.30) | 0.71 | 48.36% |
|  |  | **Control - Happy** | **HC - DEP** | 0.117 | (-0.07, 0.31) | 0.89 | 41.62% |
|  |  | **Control - Happy** | **BD - DEP** | 0.02 | (-0.25, 0.27) | 0.42 | 54.84% |
|  |  | **Control - Sad** | **HC - BD** | -0.194 | (-0.39, -0.00) | **2.97** | 16.82% |
|  |  | **Control - Sad** | **HC - DEP** | 0.047 | (-0.14, 0.24) | 0.49 | 64.02% |
|  |  | **Control - Sad** | **BD - DEP** | 0.241 | (-0.01, 0.49) | **2.39** | 13.08% |
| **Drift Rate** | **Masked** | **Control - Happy** | **HC - BD** | 0.027 | (-0.24, 0.28) | 0.61 | 53.34% |
|  |  | **Control - Happy** | **HC - DEP** | -0.141 | (-0.40, 0.12) | 1.05 | 34.3% |
|  |  | **Control - Happy** | **BD - DEP** | -0.168 | (-0.51, 0.17) | 0.89 | 27.94% |
|  |  | **Control - Sad** | **HC - BD** | 0.157 | (-0.10, 0.42) | 1.2 | 30.5% |
|  |  | **Control - Sad** | **HC - DEP** | -0.346 | (-0.60, -0.09) | 20.44 | 2.99% |
|  |  | **Control - Sad** | **BD - DEP** | -0.503 | (-0.85, -0.17) | 39.26 | 0.91% |
|  | **Unmasked** | **Control - Happy** | **HC - BD** | 0.088 | (-0.18, 0.35) | 0.74 | 45.16% |
|  |  | **Control - Happy** | **HC - DEP** | -0.128 | (-0.40, 0.13) | 0.96 | 36.88% |
|  |  | **Control - Happy** | **BD - DEP** | -0.216 | (-0.56, 0.14) | 1.18 | 21.47% |
|  |  | **Control - Sad** | **HC - BD** | 0.054 | (-0.20, 0.31) | 0.63 | 52.02% |
|  |  | **Control - Sad** | **HC - DEP** | -0.124 | (-0.37, 0.13) | 0.91 | 38.5% |
|  |  | **Control - Sad** | **BD - DEP** | -0.178 | (-0.51, 0.15) | 0.92 | 27.03% |

*Estimate: posterior mean of the prime effect on the parameter; CrI: 95% credibility interval for the posterior mean; BF₁₀: Bayes Factor in favor of the alternative hypothesis (values > 3 are shown in bold, indicating at least moderate evidence; values < 0.3 are shown in bold, indicating at least moderate evidence in favor of the null hypothesis); ROPE (%): percentage of the posterior distribution falling within the region of practical equivalence.*

## Supplementary Table S28. Drift Diffusion Modeling results for the effect of primes on starting point and drift rate between priming type across groups

| **Parameter** | **Group** | **Prime Contrast** | **Priming Type Contrast** | **Estimate** | **CrI** | **BF_10_** | **ROPE (%)** |
| --- | --- | --- | --- | --- | --- | --- | --- |
| **Starting Point** | **HC** | **Control - Happy** | **Masked - Unmasked** | -0.122 | (-0.25, 0.01) | 1.79 | 37.26% |
|  |  | **Control - Sad** | **Masked - Unmasked** | 0.198 | (0.06, 0.33) | 29.02 | 6.96% |
|  | **BD** | **Control - Happy** | **Masked - Unmasked** | -0.152 | (-0.38, 0.09) | 0.93 | 31.5% |
|  |  | **Control - Sad** | **Masked - Unmasked** | 0.136 | (-0.09, 0.35) | 0.81 | 35.77% |
|  | **DEP** | **Control - Happy** | **Masked - Unmasked** | 0.016 | (-0.21, 0.24) | 0.4 | 61.64% |
|  |  | **Control - Sad** | **Masked - Unmasked** | 0.084 | (-0.13, 0.31) | 0.52 | 50.52% |
| **Drift Rate** | **HC** | **Control - Happy** | **Masked - Unmasked** | 0.286 | (0.11, 0.46) | 59.4 | 1.97% |
|  |  | **Control - Sad** | **Masked - Unmasked** | 0.045 | (-0.13, 0.22) | 0.51 | 67.81% |
|  | **BD** | **Control - Happy** | **Masked - Unmasked** | 0.346 | (0.06, 0.63) | 8.57 | 4.66% |
|  |  | **Control - Sad** | **Masked - Unmasked** | -0.058 | (-0.34, 0.24) | 0.57 | 46.83% |
|  | **DEP** | **Control - Happy** | **Masked - Unmasked** | 0.298 | (0.01, 0.59) | 4.16 | 8.58% |
|  |  | **Control - Sad** | **Masked - Unmasked** | 0.267 | (-0.02, 0.55) | 2.71 | 12.27% |

*Estimate: posterior mean of the prime effect on the parameter; CrI: 95% credibility interval for the posterior mean; BF₁₀: Bayes Factor in favor of the alternative hypothesis (values > 3 are shown in bold, indicating at least moderate evidence; values < 0.3 are shown in bold, indicating at least moderate evidence in favor of the null hypothesis); ROPE (%): percentage of the posterior distribution falling within the region of practical equivalence.*

## Supplementary Table S29. Drift Diffusion Modeling results for the effect of mood on starting point and drift rate across groups - All groups by primes

| **Prime** | **Parameter** | **Estimate** | **CrI** | **BF_10_** | **ROPE (%)** |
| --- | --- | --- | --- | --- | --- |
| **Control** | **Starting point** | -0.01 | (-0.03, 0.01) | 0.08 | 100% |
|  | **Drift rate** | 0.116 | (0.01, 0.23) | **2.14** | 38.59% |

*Estimate: posterior mean of the prime effect on the parameter; CrI: 95% credibility interval for the posterior mean; BF₁₀: Bayes Factor in favor of the alternative hypothesis (values > 3 are shown in bold, indicating at least moderate evidence; values < 0.3 are shown in bold, indicating at least moderate evidence in favor of the null hypothesis); ROPE (%): percentage of the posterior distribution falling within the region of practical equivalence.*

## Supplementary Table S30. Drift Diffusion Modeling results for the effect of mood on starting point and drift rate across groups - All groups all primes

| **Group** | **Parameter** | **Estimate** | **CrI** | **BF_10_** | **ROPE (%)** |
| --- | --- | --- | --- | --- | --- |
| **HC** | **Starting point** | -0.02 | (-0.05, 0.02) | 0.17 | 100% |
|  | **Drift rate** | 0.142 | (0.00, 0.28) | **2.62** | 27.42% |
| **BD** | **Starting point** | -0.015 | (-0.06, 0.03) | 0.1 | 99.99% |
|  | **Drift rate** | 0.058 | (-0.14, 0.27) | 0.43 | 59.69% |
| **DEP** | **Starting point** | 0.004 | (-0.03, 0.04) | 0.07 | 100% |
|  | **Drift rate** | 0.149 | (-0.02, 0.32) | 1.33 | 28.16% |

*Estimate: posterior mean of the prime effect on the parameter; CrI: 95% credibility interval for the posterior mean; BF₁₀: Bayes Factor in favor of the alternative hypothesis (values > 3 are shown in bold, indicating at least moderate evidence; values < 0.3 are shown in bold, indicating at least moderate evidence in favor of the null hypothesis); ROPE (%): percentage of the posterior distribution falling within the region of practical equivalence.*

# Supplementary Results

## Supplementary Results S1. No significant correlation between Reactivity Index (PC3) and Reaction Time Priming Effects

For unambiguous targets (happy/sad), in HC, there was no significant correlation between RT priming effect (Incongruent - Congruent trials) and PC3 scores in masked (*r* = 0.02, *p* = .900), nor unmasked trials (*r* = 0.11, *p* = .444). Similarly, in BD patients, we found no significant relationship between RT priming effect and PC3 scores in masked (*r* = 0.46, *p* = .139) and unmasked trials (*r* = 0.53, *p* = .050). Although the Pearson coefficients were relatively high, indicating a potential trend, the sample size (n = 14) was probably insufficient to achieve adequate statistical power for detecting a significant correlation. Regarding DEP, we did not find any significant effects in masked (*r* = 0.33, *p* = .190) and unmasked trials (*r* = -0.39, *p* = .119).

For ambiguous targets (morphs), in HC, there was no significant correlation between RT priming effect (Incongruent - Congruent trials) and PC3 in masked (ρ = 0.02, *p* = .910) nor unmasked trials (ρ = 0.06, *p* = .694). Similarly, in BD, we found no significant relationship between RT priming effects and PC3 scores in masked (r = 0.33, *p* = .298) nor unmasked trials (ρ = -0.29, *p* = .344). In DEP, we did not find significant effects in masked (ρ = -0.04, *p* = .869) nor unmasked trials (*r* = -0.08, *p* = .763). See results across groups in Figure S4.

# Supplementary Figures

## Supplementary Figures S1. Loadings and group differences on PC2

*Figure S1.* *Loadings and group differences on PC2.* **A.** Items loadings on PC2, limited to those with absolute values ≥ 0.15 for clarity. Loadings represent the contribution of each item to the principal component. Items are color-coded by questionnaire (PANAS, MATHyS – Part 1, Part 2). PC2 primarily captures high emotional intensity and mixed-valence states. **B.** Group differences in PC2 scores across groups (BD, DEP, HC). Each dot represents PC2 score for one participant, and the vertical bar indicates the group mean. Violin plots display the distribution and density of scores across participants. Statistically significant effects are denoted by p < .05 (p < .05 *, p < .01 **, p < .001 ***).

## Supplementary Figures S2. Loadings and group differences on PC3

*Figure S2.* *Loadings and group differences on PC3.* **A.** Items loadings on PC3, limited to those with absolute values ≥ 0.15 for clarity. Loadings represent the contribution of each item to the principal component. Items are color-coded by questionnaire (PANAS, MATHyS – Part 1, Part 2). PC3 primarily reflects reactivity, distractibility, and hypersensitivity. **B.** Group differences in PC3 scores across groups (BD, DEP, HC). Each dot represents PC2 score for one participant, and the vertical bar indicates the group mean. Violin plots display the distribution and density of scores across participants.

## Supplementary Figures S3. Loadings and group differences on PC4

*Figure S3.* *Loadings and group differences on PC4.* **A.** Items loadings on PC4, limited to those with absolute values ≥ 0.15 for clarity. Loadings represent the contribution of each item to the principal component. Items are color-coded by questionnaire (PANAS, MATHyS – Part 1, Part 2). PC4 seemed to represent internal tension and sensory-affective excitability. **B.** Group differences in PC4 scores across groups (BD, DEP, HC). Each dot represents PC4 score for one participant, and the vertical bar indicates the group mean. Violin plots display the distribution and density of scores across participants.

## Supplementary Figures S4. Correlation between PC3 (Reactivity Index) and Reaction Time Priming Effects on unambiguous targets (happy/sad) preceded by masked primes

*Figure S4.* Correlation between PC3 scores and RT priming effects on unambiguous happy and sad targets preceded by masked primes in **A.** HC, **B.** BD, and **C.** DEP patients. Each panel represents a scatterplot of d′ scores, reflecting participants’ ability to discriminate masked happy from sad primes, and RT priming effects in milliseconds, computed as the difference in mean RTs between incongruent and congruent trials. A linear regression line is shown with 95% confidence interval shading.

## Supplementary Figures S5. Correlation between PC3 (Reactivity Index) and Reaction Time Priming Effects on unambiguous targets (happy/sad) preceded by unmasked primes

*Figure S5.* Correlation between PC3 scores and RT priming effects on unambiguous happy and sad targets preceded by unmasked primes in **A.** HC, **B.** BD, and **C.** DEP patients. Each panel represents a scatterplot of d′ scores, reflecting participants’ ability to discriminate masked happy from sad primes, and RT priming effects in milliseconds, computed as the difference in mean RTs between incongruent and congruent trials. A linear regression line is shown with 95% confidence interval shading.

## Supplementary Figures S6. Correlation between PC3 (Reactivity Index) and Reaction Time Priming Effects on ambiguous targets (morphs) preceded by masked primes

*Figure S6.* Correlation between PC3 scores and RT priming effects on ambiguous morphed targets preceded by masked primes in **A.** HC, **B.** BD, and **C.** DEP patients. Each panel represents a scatterplot of d′ scores, reflecting participants’ ability to discriminate masked happy from sad primes, and RT priming effects in milliseconds, computed as the difference in mean RTs between incongruent and congruent trials. A linear regression line is shown with 95% confidence interval shading.

## Supplementary Figures S7. Correlation between PC3 (Reactivity Index) and Reaction Time Priming Effects on ambiguous targets (morphs) preceded by unmasked primes

*Figure S7* Correlation between PC3 scores and RT priming effects on ambiguous morphed targets preceded by unmasked primes in **A.** HC, **B.** BD, and **C.** DEP patients. Each panel represents a scatterplot of d′ scores, reflecting participants’ ability to discriminate masked happy from sad primes, and RT priming effects in milliseconds, computed as the difference in mean RTs between incongruent and congruent trials. A linear regression line is shown with 95% confidence interval shading.

## Supplementary Figures S8. Posterior predictive checks and MCMC diagnostics for the Bayesian multilevel model on RTs to unambiguous targets

Top panels: observed (dark line) and simulated (light lines) RT distributions by group. Bottom panels: trace plots and posterior density overlays for key fixed effects parameters across 8 chains (Rhat < 1.01 for all parameters).

## Supplementary Figures S9. Posterior predictive checks and MCMC diagnostics for the Bayesian multilevel model on responses to ambiguous targets

Top panels: observed (dark line) and simulated (light lines) RT distributions by group. Bottom panels: trace plots and posterior density overlays for key fixed effects parameters across 8 chains (Rhat < 1.01 for all parameters).

## Supplementary Figures S10. Posterior predictive checks and MCMC diagnostics for the Bayesian multilevel model on RTs to ambiguous targets

Top panels: observed (dark line) and simulated (light lines) RT distributions by group. Bottom panels: trace plots and posterior density overlays for key fixed effects parameters across 8 chains (Rhat < 1.01 for all parameters).

## Supplementary Figures S11. Posterior predictive checks and MCMC diagnostics for the drift diffusion model on ambiguous targets

Top panels: observed (dark line) and simulated (light lines) RT distributions by group. Bottom panels: trace plots and posterior density overlays for key fixed effects parameters across 8 chains (Rhat < 1.01 for all parameters).

## Supplementary Figures S12. Correlation between *d’* happy-sad and RT priming effects in HC, BD and DEP.

*Figure S12.* Correlation between happy–sad d′ and RT priming effects, shown for unambiguous targets (top row) and ambiguous morph targets (bottom row), separately in HC, BD, and DEP patients (columns). Each panel shows a scatterplot of *d′* scores, reflecting participants' ability to discriminate masked happy from sad primes, against RT priming effects in milliseconds, computed as the difference in mean RTs between incongruent and congruent trials. The line shows the posterior median association with 95% credible interval shading, derived from a Bayesian correlation analysis; BF_10_ is reported in each panel.

# References

[Bürkner, P.-C. (2017). brms : An R Package for Bayesian Multilevel Models Using Stan. *Journal of Statistical Software*, *80*, 1‑28. https://doi.org/10.18637/jss.v080.i01](https://www.zotero.org/google-docs/?0NSodR)

[Ebner, N. C., Riediger, M., & Lindenberger, U. (2010). FACES—A database of facial expressions in young, middle-aged, and older women and men : Development and validation. *Behavior Research Methods*, *42*(1), 351‑362. https://doi.org/10.3758/BRM.42.1.351](https://www.zotero.org/google-docs/?0NSodR)

[Green, D. M., & Swets, J. A. (1966). *Signal detection theory and psychophysics* (p. xi, 455). John Wiley.](https://www.zotero.org/google-docs/?0NSodR)

Gelman, A., Hill, J., & Yajima, M. (2009). Why we (usually) don’t have to worry about multiple comparisons. arXiv. https://arxiv.org/abs/0907.2478

Hautus, M.J. Corrections for extreme proportions and their biasing effects on estimated values of d′. Behavior Research Methods, Instruments, & Computers 27, 46–51 (1995). https://doi.org/10.3758/BF03203619

[Ji, J. L., Helmer, M., Fonteneau, C., Burt, J. B., Tamayo, Z., Demšar, J., Adkinson, B. D., Savić, A., Preller, K. H., Moujaes, F., Vollenweider, F. X., Martin, W. J., Repovš, G., Cho, Y. T., Pittenger, C., Murray, J. D., & Anticevic, A. (2021). Mapping brain-behavior space relationships along the psychosis spectrum. *eLife*, *10*, e66968. https://doi.org/10.7554/eLife.66968](https://www.zotero.org/google-docs/?0NSodR)

[Lee, K., Ji, J. L., Fonteneau, C., Berkovitch, L., Rahmati, M., Pan, L., Repovš, G., Krystal, J. H., Murray, J. D., & Anticevic, A. (2024). Human brain state dynamics are highly reproducible and associated with neural and behavioral features. *PLOS Biology*, *22*(9), e3002808. https://doi.org/10.1371/journal.pbio.3002808](https://www.zotero.org/google-docs/?0NSodR)

Morey, R. D., & Rouder, J. N. (2024). BayesFactor: Computation of Bayes Factors for Common Designs.

https://CRAN.R-project.org/package=BayesFactor

[Moujaes, F., Ji, J. L., Rahmati, M., Burt, J. B., Schleifer, C., Adkinson, B. D., Savic, A., Santamauro, N., Tamayo, Z., Diehl, C., Kolobaric, A., Flynn, M., Rieser, N., Fonteneau, C., Camarro, T., Xu, J., Cho, Y., Repovs, G., Fineberg, S. K., … Anticevic, A. (2024). Ketamine induces multiple individually distinct whole-brain functional connectivity signatures. *eLife*, *13*, e84173. https://doi.org/10.7554/eLife.84173](https://www.zotero.org/google-docs/?0NSodR)

[Peirce, J., Gray, J. R., Simpson, S., MacAskill, M., Höchenberger, R., Sogo, H., Kastman, E., & Lindeløv, J. K. (2019). PsychoPy2 : Experiments in behavior made easy. *Behavior Research Methods*, *51*(1), 195‑203. https://doi.org/10.3758/s13428-018-01193-y](https://www.zotero.org/google-docs/?0NSodR)

Vehtari, A., Gelman, A. & Gabry, J. Practical Bayesian model evaluation using leave-one-out cross-validation and WAIC. Stat Comput 27, 1413–1432 (2017). https://doi.org/10.1007/s11222-016-9696-4

[Wagenmakers, E.-J. (2007). A practical solution to the pervasive problems ofp values. *Psychonomic Bulletin & Review*, *14*(5), 779‑804. https://doi.org/10.3758/BF03194105](https://www.zotero.org/google-docs/?0NSodR)

[Wagenmakers, E.-J., Marsman, M., Jamil, T., Ly, A., Verhagen, J., Love, J., Selker, R., Gronau, Q. F., Šmíra, M., Epskamp, S., Matzke, D., Rouder, J. N., & Morey, R. D. (2018). Bayesian inference for psychology. Part I : Theoretical advantages and practical ramifications. *Psychonomic Bulletin & Review*, *25*(1), 35‑57. https://doi.org/10.3758/s13423-017-1343-3](https://www.zotero.org/google-docs/?0NSodR)
